# Protein arginine methyltransferase 1 regulates B cell fate after positive selection in the germinal center in mice

**DOI:** 10.1084/jem.20220381

**Published:** 2023-06-13

**Authors:** Ludivine C. Litzler, Astrid Zahn, Kiersten L. Dionne, Adrien Sprumont, Silvana R. Ferreira, Michael R.F. Slattery, Stephen P. Methot, Anne-Marie Patenaude, Steven Hébert, Nisha Kabir, Poorani Ganesh Subramani, Seolkyoung Jung, Stéphane Richard, Claudia L. Kleinman, Javier M. Di Noia

**Affiliations:** 1 Institut de Recherches Cliniques de Montréal, Montreal, Canada; 2 Department of Microbiology and Immunology, McGill University, Montreal, Canada; 3 Lady Davis Institute for Medical Research, Montreal, Canada; 4 Department of Human Genetics, McGill University, Montreal, Canada; 5 Department of Medicine, McGill University, Montreal, Canada; 6 Biodata Mining and Discovery Section, National Institute of Arthritis and Musculoskeletal and Skin Diseases, National Institutes of Health, Bethesda, MD, USA; 7 Gerald Bronfman Departments of Oncology, McGill University, Montreal, Canada; 8 Department of Biochemistry, McGill University, Montreal, Canada; 9 Department of Medicine, Université de Montréal, Montreal, Canada

## Abstract

Positively selected germinal center B cells (GCBC) can either resume proliferation and somatic hypermutation or differentiate. The mechanisms dictating these alternative cell fates are incompletely understood. We show that the protein arginine methyltransferase 1 (Prmt1) is upregulated in murine GCBC by Myc and mTORC-dependent signaling after positive selection. Deleting *Prmt1* in activated B cells compromises antibody affinity maturation by hampering proliferation and GCBC light zone to dark zone cycling. Prmt1 deficiency also results in enhanced memory B cell generation and plasma cell differentiation, albeit the quality of these cells is compromised by the GCBC defects. We further demonstrate that Prmt1 intrinsically limits plasma cell differentiation, a function co-opted by B cell lymphoma (BCL) cells. Consistently, PRMT1 expression in BCL correlates with poor disease outcome, depends on MYC and mTORC1 activity, is required for cell proliferation, and prevents differentiation. Collectively, these data identify PRMT1 as a determinant of normal and cancerous mature B cell proliferation and differentiation balance.

## Teaser

Protein arginine methyltransferase 1 (PRMT1) is upregulated via Myc and mTORC1 during positive selection and influences germinal center B cell fate. PRMT1 promotes antibody affinity maturation by favoring dark zone fate and proliferation while limiting differentiation, two functions that are co-opted by mature B cell lymphoma cells.

## Introduction

High-affinity antibody responses are generated in the germinal centers (GC) in lymphoid tissue (Victora and Nussenzweig, 2022). Naive follicular B cells that recognize cognate antigens and interact with primed T cells become activated and can then join GCs. The GC is organized into two anatomical regions, the dark (DZ) and light (LZ) zones (Victora and Nussenzweig, 2022). GC B cells (GCBC) in the DZ proliferate and undergo somatic hypermutation (SHM), which changes the antibody affinity. The transit from the DZ to the LZ permits interactions between GCBC and T follicular helper (T_fh_) cells, enabling positive selection of those GCBC bearing higher affinity antibodies (Cyster and Allen, 2019; Shlomchik et al., 2019; Victora and Nussenzweig, 2022). Positively selected GCBC are characterized by transient Myc expression (Calado et al., 2012; Dominguez-Sola et al., 2012) and induced by the B cell receptor (BCR) and T cell–mediated CD40 signaling in LZ B cells (Luo et al., 2018; Cyster and Allen, 2019). Positive selection promotes DZ reentry, proliferation, and SHM (Victora et al., 2010; Gitlin et al., 2014). However, Myc^+^ cells are heterogenous and contain plasma cell (PC) and memory B cell (MBC) precursors in addition to DZ reentrants, implying the existence of additional mechanisms to impart distinct fates to positively selected B cells (Suan et al., 2017; Inoue et al., 2021; Toboso-Navasa et al., 2020; Ise et al., 2018; Nakagawa et al., 2021). These mechanisms are incompletely understood, but higher mTORC1 signaling, characteristic of cells entering the DZ, and regulation of the MYC transcriptional activity could explain some of this diversity (Ersching et al., 2017; Toboso-Navasa et al., 2020). PC also tend to have high-affinity BCR while most MBC display low-affinity BCR, with CD40 signaling strength further regulating the formation of both (Phan et al., 2006; Shinnakasu et al., 2016; Inoue et al., 2017; Suan et al., 2017; Ise et al., 2018; Koike et al., 2019; Viant et al., 2020).

The posttranslational methylation of protein arginine residues regulates many cellular processes (Xu and Richard, 2021). Most protein arginine methyltransferases (PRMTs) transfer two methyl groups. Depending on whether they add the two methyl groups to the same or a different nitrogen atom of the arginine guanidino group, PRMTs are respectively classified as type I, producing asymmetrical dimethyl arginine (aDMA), or type II, producing symmetrical DMA, which have distinct effects on protein function (Yang and Bedford, 2013; Xu and Richard, 2021). Protein arginine methylation is dynamic and relevant in lymphocytes, but remains poorly studied in B cells (Ying et al., 2015; Hata and Mizuguchi, 2013; Infantino et al., 2010, 2017; Dolezal et al., 2017; Litzler et al., 2019; Geoghegan et al., 2015). PRMT1 is the main enzyme catalyzing aDMA modification in a large repertoire of substrates, thus regulating transcription, DNA repair, and signaling, among other processes (Xu and Richard, 2021). Type I and II PRMT inhibitors are promising cancer therapies, effective in preclinical tumor models and currently in clinical trials (Wu et al., 2021). Type I PRMT inhibitors, which mainly target the predominant PRMT1 in vivo, induce cell death and arrest proliferation in leukemia and lymphoma cell lines and reduce their ability to form tumors in xenotransplantation models (Fedoriw et al., 2019; Fong et al., 2019). As these compounds go into clinical trials for GC-derived B cell lymphoma (BCL; Wu et al., 2021), it is important to define the normal function of PRMT1 in GCBC, to understand their mechanism of action.

Conditional ablation during B cell development has shown that PRMT1 promotes the differentiation of pre-B cells (Infantino et al., 2010; Dolezal et al., 2017; Hata et al., 2016). Echoing the multiplicity of PRMT1 substrates, this is achieved by at least two mechanisms. First, aDMA at the Igα/CD79A dampens PI3K/AKT signaling from the pre-BCR, thus favoring differentiation (Infantino et al., 2010). Second, CDK4 methylation causes G1 arrest by disrupting the interaction with cyclin D3 to allow *IgL* rearrangement (Dolezal et al., 2017). The function of PRMT1 in mature B cells is poorly understood. Deleting *Prmt1* from the pro-B (Hata et al., 2016) or transitional B cell (Infantino et al., 2017) stages in mice has shown normal or reduced T cell–dependent antibody responses, respectively. These reports also found opposite effects on the proliferation of PRMT1-deficient mature B cells stimulated ex vivo (Hata et al., 2016; Infantino et al., 2017). These discrepant observations might be explained by differences in excision efficiency and compensatory mechanisms selected during the ontogeny of PRMT1-null B cells. Nonetheless, they call for further scrutiny to clarify the role of PRMT1 in mature B cells. Moreover, the role of PRMT1 in GCBC cannot be gleaned from those systems because PRMT1-deficient B cells show activation defects, including increased apoptosis (Infantino et al., 2017), which would compromise GC formation. Accordingly, we have shown that PRMT5 protects B cells from apoptosis during activation, but is dispensable for the survival of GCBC (Litzler et al., 2019).

Here, we analyzed the function of PRMT1 in mice after B cell activation. We find that Prmt1 is required for GC expansion and the normal GC dynamics underpinning high-affinity antibody responses. This is achieved by preventing premature differentiation of activated B cells and by favoring GCBC DZ reentry instead of MBC fate. Accordingly, we show that *Prmt1* is a direct Myc target that is upregulated in positively selected cells, which also requires mTORC1 activity. Moreover, *PRMT1* expression correlates with poor outcome in BCL patients and is also expressed in a MYC- and mTORC1-dependent manner in BCL cells. PRMT1 deletion or type I PRMT inhibition in activated or BCL cells causes PC differentiation, providing new insights into the mechanisms by which PRMT1 inhibition would be effective against BCL.

## Results

### Predominance of PRMT1 expression in activated and GCBC

**Figure 1.**
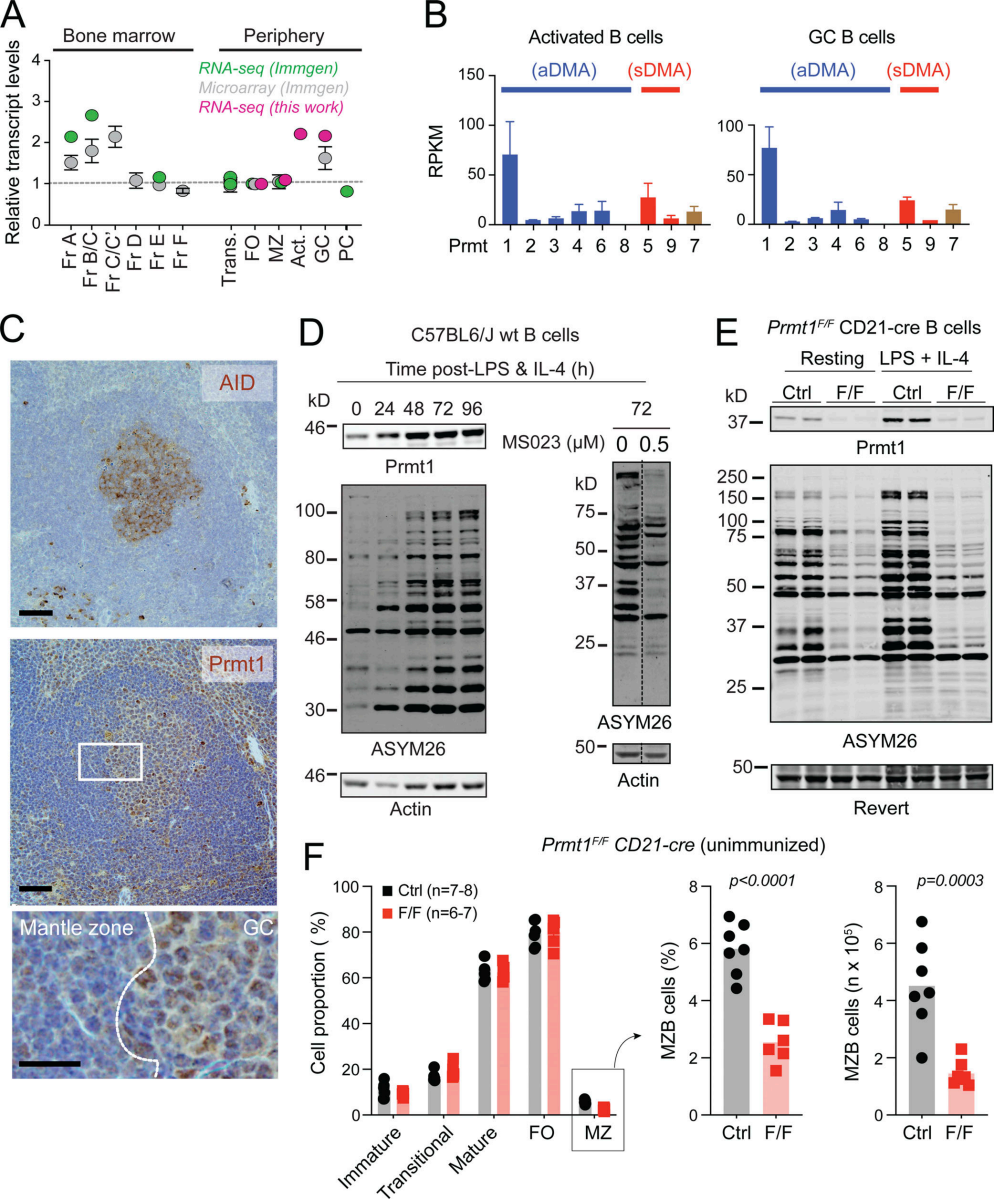
**Prmt1 expression in activated and GCBC. (A)** Prmt1 transcript levels in three mouse B cell datasets, each normalized to follicular (FO) B cells. Fr, Hardy’s fractions of B cell development; Trans, transitional; MZ, marginal zone; Act., ex vivo-activated mouse splenic B cells (50 μg/ml LPS + 2.5 ng/ml IL-4, 72 h). **(B)** PRMT transcript levels in activated (as in A) and in GCBC sorted from lymph nodes of immunized mice. Average + SEM RPKM from two biological replicates. **(C)** Immunohistochemistry for Prmt1, and AID as GC marker, on consecutive spleen sections from immunized mice. Representative of two mice/genotype independently analyzed. Scale bars, 100 µm (top, middle) and 20 µm (bottom). **(D)** Western blot of PRMT1, aDMA-modified proteins (ASYM26), and actin in extracts of resting and stimulated splenic B cells. MS023 = inhibitor of type I PRMTs. **(E)** Prmt1 and aDMA-proteins in extracts of resting or activated splenic B cells from two CD21-cre (Ctrl) and two *Prmt1*^*F/F*^ CD21-cre (F/F) mice. Revert protein stain as the loading control. **(F)** Proportion of splenic B cell subpopulations in individual *Prmt1*^*F/F*^ *CD21-cre* (F/F) and *CD21-cre* (Ctrl) mice (symbols) from three independent experiments, with bars indicating means. MZB cell numbers are presented. P values by unpaired, two-tailed Student’s *t* test are indicated in the figure. Source data are available for this figure: SourceData F1.

**Figure S1.**
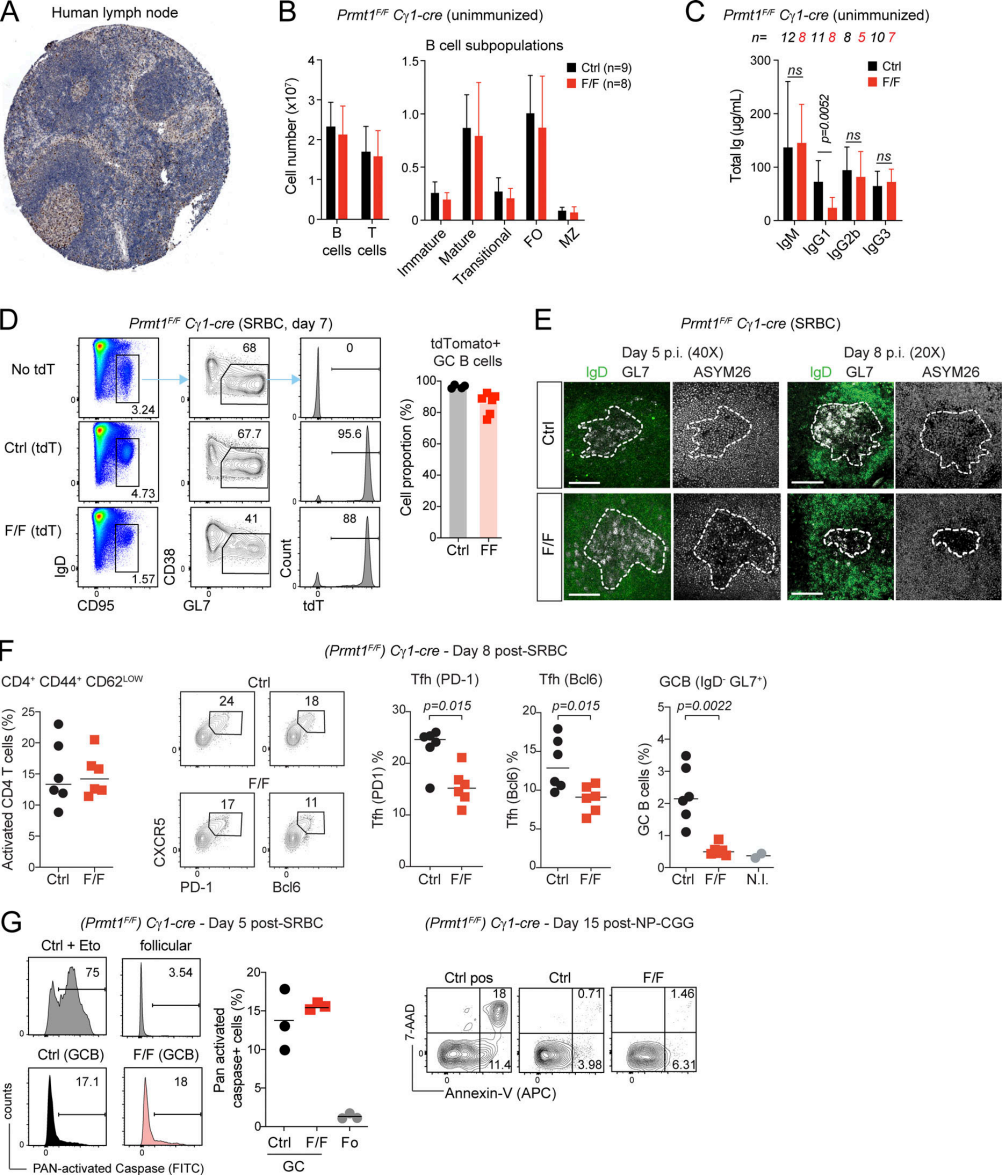
**Characterization of *Prmt1***^***F/F***^ **Cγ1-cre mice B cells.** Related to Figs. 1, 2, and 3*.* **(A)** PRMT1 immunohistochemistry in human lymph node stained with antibody CAB022550, as obtained from the Human Protein Atlas Database. (Image credit: Human Protein Atlas, proteinatlas.org; https://www.proteinatlas.org/ENSG00000126457-PRMT1/tissue/lymph+node#img). **(B)** Mean + SD number of lymphocytes and B cell subpopulations per spleen in Cγ1-cre (Ctrl) *Prmt1*^*F/F*^ Cγ1-cre (F/F) mice from three experiments. **(C)** Mean + SD levels of antibody isotypes in the pre-immune sera from Cγ1-cre (Ctrl) or *Prmt1*^*F/F*^ Cγ1-cre (F/F) mice measured by ELISA from ≥2 experiments. **(D)** Representative flow cytometry and mean proportion of tdTomato^+^ GCBCs (B220^+^ IgD^−^ CD95^+^ CD38^−^ Gl7^+^) in four Cγ1-cre R26td^Tomato^ (Ctrl) and six *Prmt1*^*F/F*^ Cγ1-cre R26td^Tomato^ (F/F) mice from two experiments. **(E)** Confocal images of immunofluorescence with antibodies against aDMA (ASYM26) in mouse splenic sections at days 5 or 8 after immunization. Dashed lines delimit GC position (IgD^−^ Gl7^+^). Scale bars, 100 µm. Representative of ≥2 mice per time point. **(F)** Activated CD4^+^ and T_fh_ cells (estimated by Cxcr5 and PD1 or Bcl6 expression) in individual (symbols) Cγ1-cre (Ctrl) or *Prmt1*^*F/F*^ Cγ1-cre (F/F) mice 8 d after SRBC immunization, from two experiments. The rightmost panel shows the proportion of GCBC in the same mice. Lines indicate mean values. N.I., non-immunized. **(G)** Apoptosis in GCBCs (B220^+^ CD95^+^ GL7^+^) from Cγ1-cre (Ctrl) or *Prmt1*^*F/F*^ Cγ1-cre (F/F) mice measured by staining with a fluorescent pan-caspase inhibitor CaspGlow (left) or Annexin V (right) in two independent experiments. Representative flow cytometry of activated by pan-caspase staining and proportion of activated caspase^+^ cells in GCBC from individual mice (symbols), with mean (lines). The positive gate for the CaspGlow experiment was set using etoposide (3 μM) to induce apoptosis. **(B, C, and F)** P values by unpaired, two-tailed Student’s *t* test are indicated in the figure.

To identify the B cell subsets in which PRMT1 might be more relevant, we performed RNA sequencing (RNA-seq) on mature B cell subsets and complemented these results with data available in the Immgen database (Heng et al., 2008). *Prmt1* mRNA levels were high during early B cell development and then peaked again in activated and GCBC (Fig. 1 A). *Prmt1* was the most highly expressed PRMT in activated and GCBC (Fig. 1 B). Immunohistochemistry confirmed higher PRMT1 protein expression in GC than follicular B cells in mouse spleen (Fig. 1 C) and human lymph node (Fig. S1 A). Consistently, PRMT1 and aDMA-modified proteins increased after activating mouse splenic B cells with LPS and IL-4 (Fig. 1 D). The type I PRMT inhibitor MS023 (Eram et al., 2016) greatly reduced the number of aDMA-modified proteins, yielding a pattern similar to B cells from *Prmt1*^*F/F*^ CD21-cre mice, which lack Prmt1 in mature B cells (Fig. 1 E; Kraus et al., 2004). Unimmunized *Prmt1*^*F/F*^ CD21-cre mice showed a reduced proportion of marginal zone B (MZB) cells but no defect in follicular B cells (Fig. 1 F), in line with previous reports (Hata et al., 2016; Infantino et al., 2017). Thus, Prmt1 is dispensable for follicular B cells but necessary for MZB cell homeostasis, and it is the predominant type I PRMT in activated and GCBC.

### PRMT1 is required after B cell activation for antibody affinity maturation

To study the role of PRMT1 in GCBC without the confounding defects in B cell activation, we used the Cγ1-cre driver to delete *Prmt1* after activation (Casola et al., 2006). *Prmt1*^*F/F*^ Cγ1-cre mice had normal numbers of T lymphocytes and resting B cell subsets (Fig. S1 B), but basal serum IgG1 was specifically reduced (Fig. S1 C), indicating an intrinsic defect in antigen-experienced B cells. A R*osa26-flox-stop-flox-tdTomato* (*R26*^*tdTomato*^) allele that permanently labels Cre^+^ cells showed that ∼86% of GCBC expressed the Cre recombinase in *Prmt1*^*F/F*^ *R26*^*tdTomato*^ Cγ1-cre mice (Fig. S1 D), suggesting efficient deletion of *Prmt1*. Accordingly, the ASYM26 signal was strongly reduced in most *Prmt1*^*F/F*^ Cγ1-cre GC cells when assayed by immunofluorescence (IF; Fig. S1 E). Some residual reactivity of the ASYM26 antibody is expected because B cells express other type I PRMTs that produce aDMA epitopes (Fig. 1 B), but the large decrease was consistent with Prmt1 being the predominant PRMT activity (Infantino et al., 2017) and showed that the GC in *Prmt1*^*F/F*^ Cγ1-cre were not made of deletion escapees.

**Figure 2.**
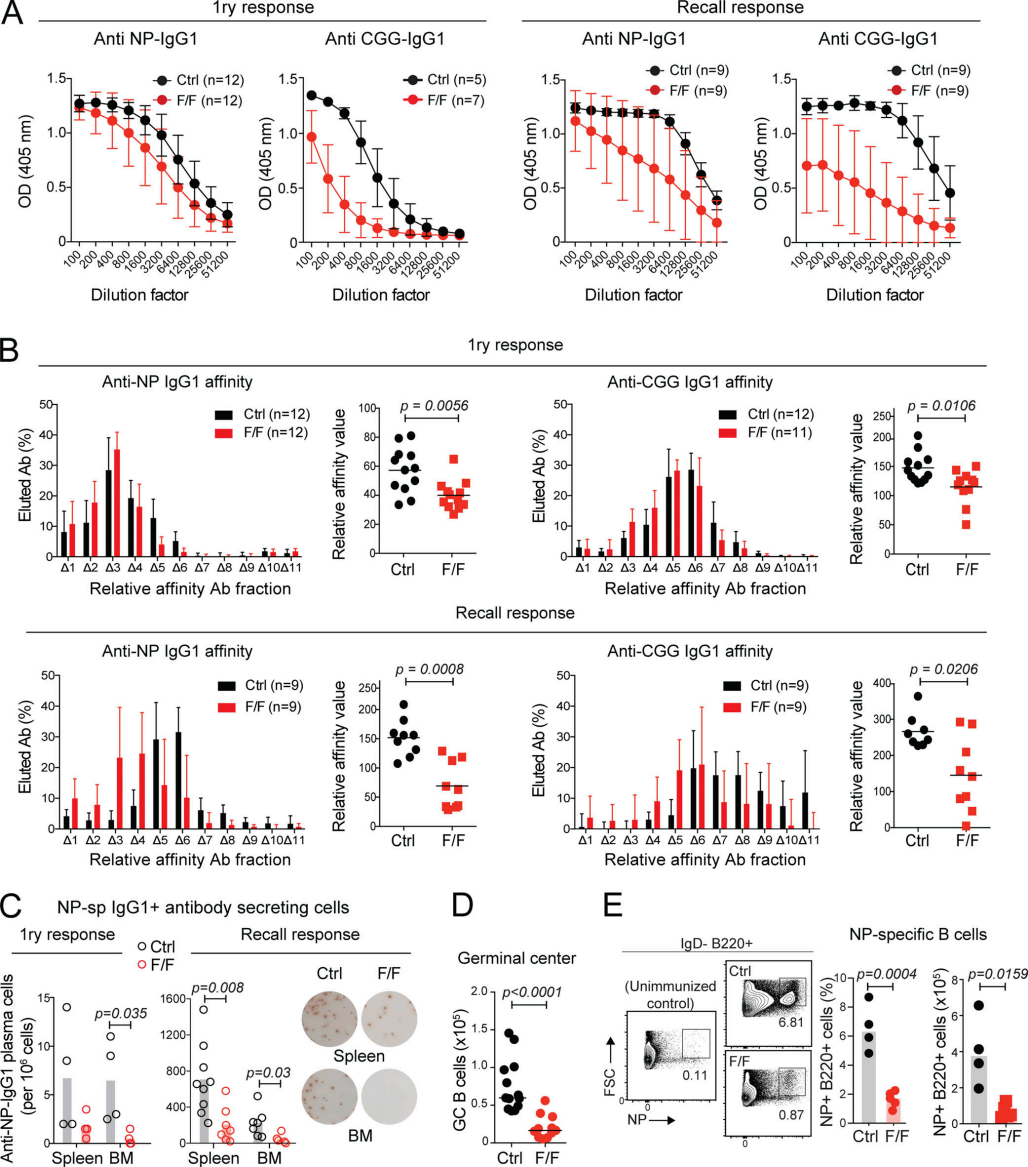
**PRMT1 is necessary for affinity maturation.** Antibody response in Cγ1-cre (Ctrl) or *Prmt1*^*F/F*^ Cγ1-cre (F/F) mice at day 14 after immunizations with NP-CGG (1ry response) or at day 7–9 after a boost given 18 wk after immunization (recall response). **(A)** Total anti-NP (NP_20_) and anti-CGG IgG1 measured by ELISA. Mean ± SD of OD values over serum dilution factor compiled from three to five independent experiments. **(B)** Affinity distribution of antigen-specific IgG1 measured by NaSCN displacement ELISA on NP_20_-BSA- or CGG-coated plates plotting mean + SD IgG1 fraction eluted at each NaSCN concentration (Δn), from three to four independent experiments. Calculated relative affinity values are plotted for individual mice (symbols) with means (bars). **(C)** Number of NP-specific IgG1 antibody-secreting cells by ELISPOT (captured on NP_20-30_-BSA) in the spleen and BM of individual mice (dots), with means (bars), from two experiments. Representative images for the recall response are shown. **(D)** Absolute number of GCBC (B220^+^ GL7^high^ CD95^+^) on day 14 after immunization in individual mice (dots) with means (bars), from three experiments. **(E)** Representative flow cytometry plots and compiled frequency and absolute numbers of antigen-specific B cells (NP^+^ IgD^−^ B220^+^) from three experiments. Non-immunized mice (left) were used to define the NP^+^ gate. **(B–E)** P values by unpaired, two-tailed Student’s *t* test are indicated in the figure.

To assess the antibody response, we immunized mice with 4-hydroxy-3-nitrophenyl conjugated to chicken gamma globulin (NP-CGG). *Prmt1*^*F/F*^ Cγ1-cre mice produced lower antigen-specific IgG1 titers than controls at day 14; the defect being modest for anti-NP but substantial for IgG1 against the more complex CGG protein mix carrier (Fig. 2 A). Both anti-NP and anti-CGG IgG1 were more severely reduced in *Prmt1*^*F/F*^ Cγ1-cre mice after a recall immunization 18 wk later (Fig. 2 A). We assessed affinity maturation by NaSCN-displacement ELISA (Zahn et al., 2013; Luxton and Thompson, 1990). The affinity of both anti-CGG and anti-NP IgG1 was significantly reduced in *Prmt1*^*F/F*^ Cγ1-cre versus control mice, with the defect being larger in the recall than the primary response (Fig. 2 B). Since the recall response in this immunization model is dominated by high-affinity MBC (Mesin et al., 2020), this suggested a defective MBC compartment (see below). Consistent with the IgG1 titers defect, *Prmt1*^*F/F*^ Cγ1-cre mice produced fewer NP-specific IgG1^+^ antibody-secreting cells than controls in the spleen and BM at either time (Fig. 2 C). The number of GCBC after immunization was 3.6-fold lower in *Prmt1*^*F/F*^ Cγ1-cre than in control mice (Fig. 2 D), indicating a GC function defect. In addition, *Prmt1*^*F/F*^ Cγ1-cre mice generated a smaller proportion and number of NP-binding cells, consistent with the defects in GC size and affinity maturation (Fig. 2 E).

We conclude that PRMT1 expression in activated B cells is necessary for the switched antibody response and affinity maturation, as well as for an optimal GC reaction.

### PRMT1 is required for GC expansion

**Figure 3.**
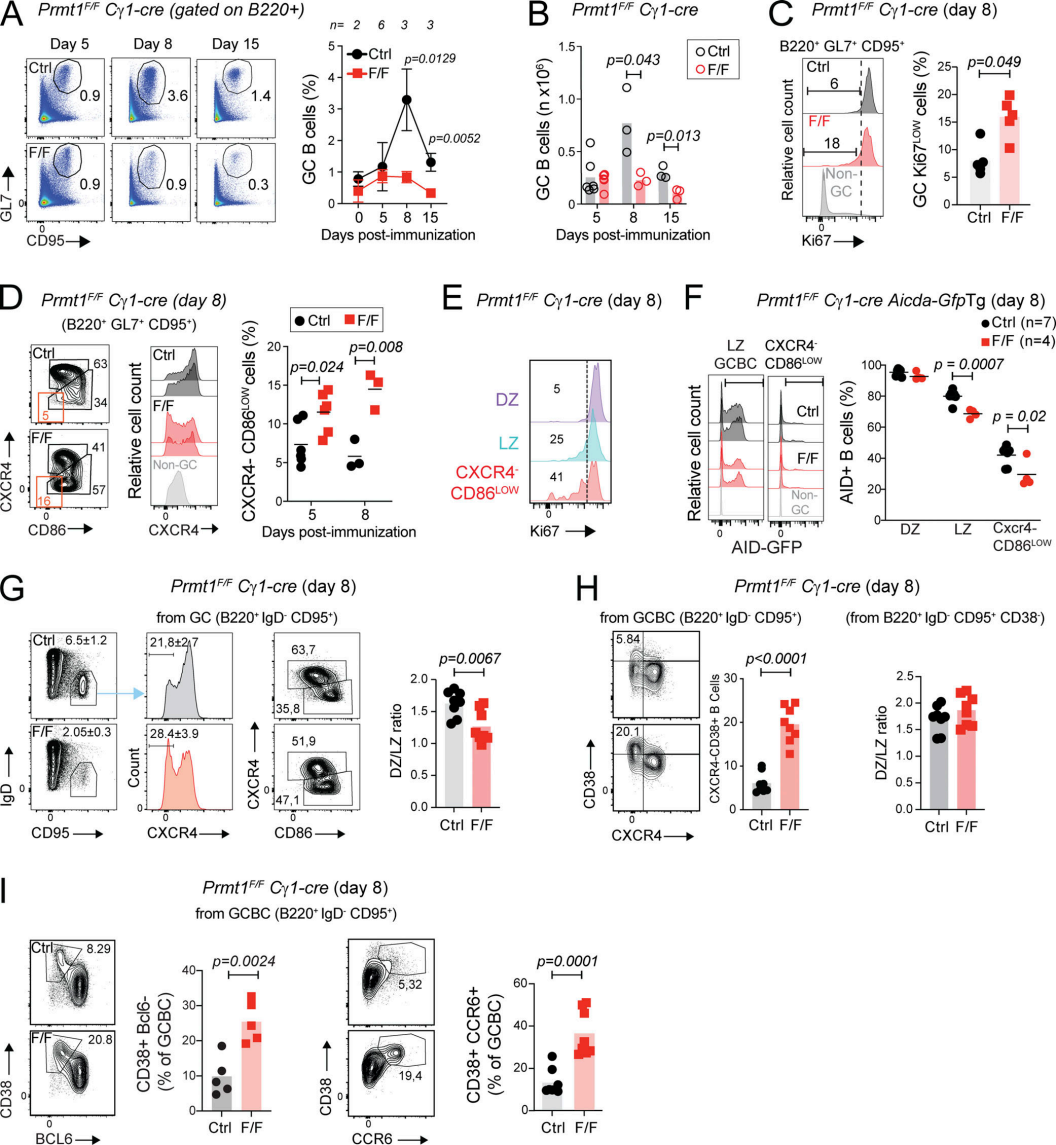
**PRMT1 is required for GC expansion.** Cγ1-cre (Ctrl) and *Prmt1*^*F/F*^ Cγ1-cre (F/F) mice analyzed at various times after immunization with SRBC. When indicated, an *Aicda*-GFPtg AID reporter transgene was used. **(A)** Representative flow cytometry and mean ± SD (except at day 0, SEM) GCBC proportion in splenocytes, for *n* mice/genotype from five experiments. **(B)** Absolute GCBC numbers compiled from two experiments, from A. **(C)** Representative flow cytometry of Ki67 staining and GC Ki67^LOW^ cell proportion compiled from two experiments. **(D)** Representative flow cytometry plots distinguishing DZ (Cxcr4^+^ CD86^LOW^) and LZ (Cxcr4^−^ CD86^HIGH^) GCBC and histograms of Cxcr4 expression, with proportion of Cxcr4^−^CD86^LOW^ cells compiled from two experiments. **(E)** Representative flow cytometry of Ki67 staining in DZ, LZ and Cxcr4^−^CD86^LOW^ GCBC, noting Ki67^LOW^ cell proportions (from C). **(F)** Representative flow cytometry of AID expression in GC LZ B cells (B220^+^ GL7^+^ CD95^+^ Cxcr4^−^ CD86^LOW^) and its Cxcr4^−^CD86^LOW^ subset, with proportion AID^+^ B cells in the DZ, LZ, and Cxcr4^−^ CD86^LOW^ cells compiled from two experiments. **(G)** Representative flow cytometry of GCBC (gated as B220^+^ IgD^−^ CD95^+^) and their Cxcr4 histogram, indicating mean ± SD proportions for eight mice from three experiments. Representative DZ/LZ gating with compiled DZ/LZ ratio plots are also shown. **(H)** Representative flow cytometry of CD38 and Cxcr4 expression in GCBC (B220^+^ IgD^−^ CD95^+^) and compiled proportion of Cxcr4^−^ CD38^+^ cells and DZ/LZ ratios after excluding CD38^+^ cells. **(I)** Representative flow cytometry and proportions of GCBC with CD38^+^ Bcl6^−^ and CD38^+^ CCR6^+^ phenotypes. **(H and I)** Same mice as in G. **(B–D and F–I)** Plots show values for individual mice (symbols) and means (bars or lines). Significant (α = 0.05) P values by unpaired, two-tailed Student’s *t* test are indicated in the figure.

To pinpoint the underlying GC defects in *Prmt1*^*F/F*^ Cγ1-cre mice, we analyzed GC kinetics. Following immunization with sheep red blood cells (SRBC), GCBC frequency (B220^+^ GL7^+^ CD95^+^) and number were similar on day 5 but significantly reduced in *Prmt1*^*F/F*^ Cγ1-cre mice on days 8 and 15 after immunization (Fig. 3, A and B). T_fh_ cells were only reduced by ∼1.5-fold, most likely a consequence of the ∼4-fold decreased GCBC in the same mice (Fig. S1 F). *Prmt1*^*F/F*^ Cγ1-cre mice showed a similar proportion of apoptotic cells than controls (Fig. S1 G) but had significantly more B cells with low Ki67 staining (Fig. 3 C). When gated as GL7^+^ CD95^+^ B cells, the GC gate in *Prmt1*^*F/F*^ Cγ1-cre mice showed an apparent reduction in DZ/LZ ratio, largely due to the accumulation of Cxcr4^−^ B cells in the LZ gate (Fig. 3 D). This Cxcr4–CD86^low^ population contained the largest proportion of Ki67^low^ cells (Fig. 3 E) and was also responsible for the reduced proportion of activation-induced deaminase (AID)^+^ B cells in the LZ of *Prmt1*^*F/F*^ Cγ1-cre compared with control mice (Fig. 3 F), as judged by an *Aicda-gfp* reporter allele (Crouch et al., 2007). To further characterize the Cxcr4^−^ B cells within the GC gate in *Prmt1*^*F/F*^ Cγ1-cre mice, we stained for IgD and CD38 to exclude potential contamination with naive or pre-GC (IgD^+^ CD38^+^) B cells. Gating GC as IgD^−^ CD95^+^ still showed the accumulation of Cxcr4^−^ B cells in the LZ gate of *Prmt1*^*F/F*^ Cγ1-cre mice (Fig. 3 G). Most of the Cxcr4^−^ B cells in the IgD^−^ CD95^+^ gate were also CD38^+^ in *Prmt1*^*F/F*^ Cγ1-cre mice, and accordingly, excluding CD38^+^ cells eliminated the DZ/LZ imbalance (Fig. 3 H). However, these cells were Bcl6^−^ and Ccr6^+^ (Fig. 3 I), suggesting an MBC phenotype that is further analyzed in a section below. We conclude that PRMT1 in activated B cells is critical for GC expansion and limits the proportion of an MBC-like population.

### Prmt1 is upregulated in positively selected GCBC

**Figure 4.**
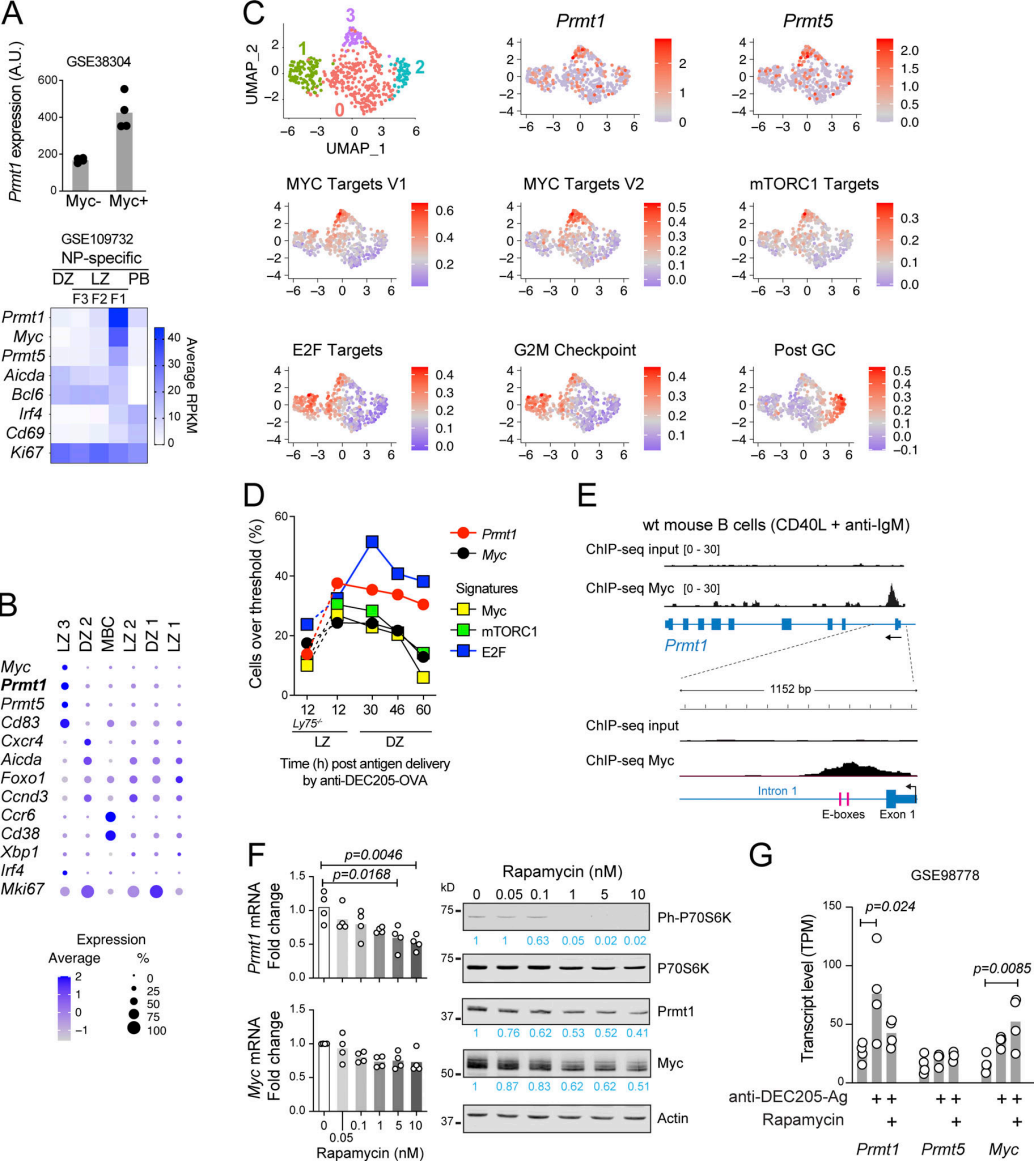
**PRMT1 is upregulated in positively selected GCBC. (A)** Top: *Prmt1* transcript levels in GCBC subsets sorted based on the expression of a *Myc* reporter (symbols are replicates; Dominguez-Sola et al., 2012). Bottom: Antigen-specific (NP-binding) B cells from DZ, three LZ fractions (F1-3), and plasmablasts (PB). Average of three replicates per fraction (Ise et al., 2018). **(B)** Relative expression of selected genes in B cell clusters obtained from scRNA-seq of splenic GCBC sorted from mice acutely infected with LCMV (GSE148805; Laidlaw et al., 2020). **(C)** Unsupervised clustering of the cells from B that expressed Myc (*n* = 466) visualized by UMAP (top left), and relative expression of genes and ssGSEA enrichment for selected signatures in the same cells. **(D)** Proportion of GCBC with above-threshold expression of the indicated genes or enrichment in selected signatures, at various timepoints after targeted antigen delivery (scRNA-seq, GSE162182; Pae et al., 2021). *Ly75*^*−/−*^ cells did not receive antigen. The assigned location of the cells (LZ or DZ) is as per the original report. **(E)** ChIP-seq tracks (GSE80669) of input and Myc signal at *Prmt1* in C57BL6 mouse splenic B cells stimulated with anti-IgM (48 h) and then CD40L on feeder cells (24 h). The position of two predicted Myc-binding sites (E-boxes) is shown. **(F)** Transcript levels by RT-qPCR (normalized to *Actin*) in purified mouse splenic B cells activated with LPS + IL-4 for 48 h before adding rapamycin for 24 h. Values for individual mice (symbols) and means (bars) from two experiments. A representative Western blot of the indicated proteins in whole cell extracts confirms mTORC1 inhibition by reduced phosphorylation of its target P70S6k. **(G)** Gene expression from bulk RNA-seq of DEC205^+^ GCBC sorted before or after delivery of antigen (anti-DEC205-Ag), alone or in combination with rapamycin treatment (Ersching et al., 2017). Values for replicates (dots) and means (bars). **(F and G)** Significant (α = 0.05) P values by one-way ANOVA with post-test are indicated in the figure. Source data are available for this figure: SourceData F4.

**Figure S2.**
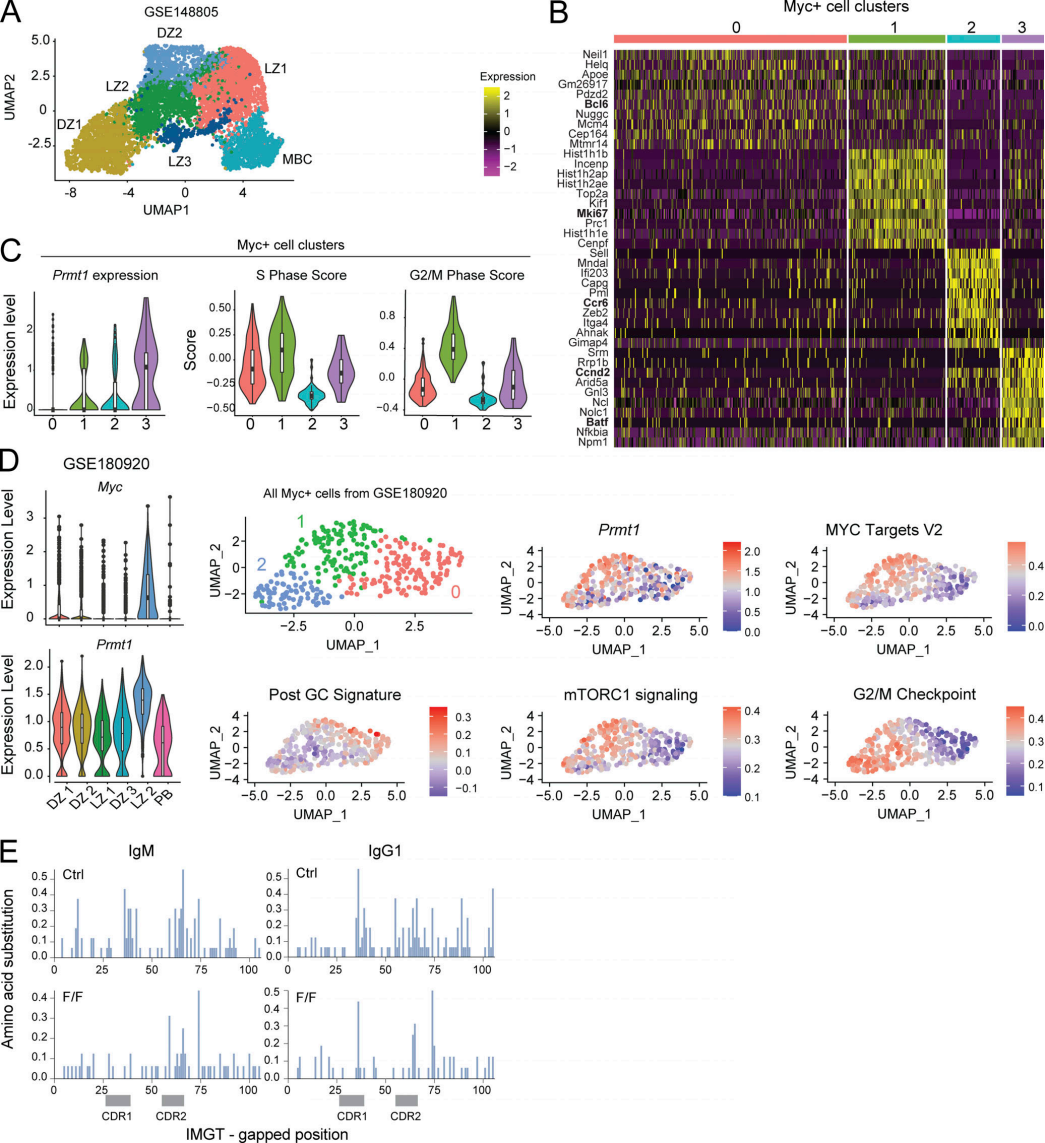
**Single cell analysis of *Prmt1* expression in GCBC.** Related to Figs. 4 and 5. **(A)** UMAP visualization of scRNA-seq data corresponding to splenic GCBC and MBCs sorted from a pool of four mice 11 d after LCMV infection (GSE148805). Single cells were clustered and colored accordingly. Clustering of GCBC subsets generally reproduced the assignments in the original report (Laidlaw et al., 2020; see Materials and methods). **(B)** Heatmap of the top 10 differentially expressed genes in four subpopulations (clusters 0–3) resulting from the unsupervised clustering of all cells in A that expressed above-threshold *Myc* transcript. **(C)** Prmt1 expression and cell cycle score for each of the Myc^+^ clusters from B). **(D)** Violin plots of *Myc* and *Prmt1* expression in the different GCBC clusters obtained as in A from the GSE180920 dataset (Duan et al., 2021). Unsupervised clustering of the cells from the dataset that expressed above-threshold *Myc* (*n* = 324), and relative expression of genes and ssGSEA enrichment for selected signatures in the same cells. **(E)** Distribution of amino acid substitution frequencies along the IgVH1-72 protein sequence in sorted GCBC from *Prmt1*^*F/F*^ Cγ1-cre (FF) and Cγ1-cre (Ctrl) mice.

While Prmt1 was expressed in most GCBC (Fig. 1 C), we analyzed its transcript levels in gene expression datasets that discriminated GCBC subsets (Dominguez-Sola et al., 2012; Ise et al., 2018; Victora et al., 2010; Victora et al., 2012) to look for a subset where it might be differentially expressed. *Prmt1* transcripts were higher in the LZ than in DZ B cell pools (147.8 vs. 82.1 reads per kilobase per million mapped reads [RPKM], respectively, GSE127267). Moreover, within the LZ, *Prmt1* was substantially upregulated in GCBC LZ subsets with high *Myc* expression (Fig. 4 A). To derive a higher-resolution picture of *Prmt1* expression, we analyzed single-cell RNA-seq (scRNA-seq) datasets of GCBC sorted from mice acutely infected with lymphocytic choriomeningitis virus (LCMV; Laidlaw et al., 2020). The B cell cluster with the highest *Prmt1* expression coincided with the highest expression of Myc (Fig. 4 B; see Fig. S2 A for clustering). Since Myc identifies B cells recently selected by T_fh_ cells (Pae et al., 2021; Dominguez-Sola et al., 2012; Calado et al., 2012), we further analyzed a potential link between Prmt1 and positive selection. We isolated the B cells with detectable Myc transcript expression (*N* = 466) from the scRNA-seq dataset and performed unsupervised clustering, which yielded four clusters (Fig. 4 C). The analysis of differentially expressed genes, cell cycle score, and enriched signatures in each cluster indicated that clusters 0, 1, and 3 were LZ GCBC subpopulations (Fig. 4 C and Fig. S2 B), while cluster 2 cells were MBC precursors exiting the GC, as indicated by low S and G2/M scores (Fig. S2 C), high post-GC signature (Fig. 4 C), and *Ccr6* expression (Fig. S2 B). Cluster 0 cells were classified as LZ based on their high *Bcl6* expression and low proliferation markers, while cluster 1 were assigned as B cells reentering the DZ based on their differential expression of *Mki67* and enrichment in S, G2/M, and E2F signatures (Fig. 4 C and Fig. S2, B and C). Cluster 3 was assigned as cells at or soon after positive selection based on the differential expression of *Batf* and *Ccnd2* (Fig. S2 B), and enrichment in Myc targets and mTORC1 activation signatures (Fig. 4 C; Pae et al., 2021; Dominguez-Sola et al., 2012; Calado et al., 2012; Inoue et al., 2017). *Prmt1* was among the differentially expressed genes distinguishing cluster 3 (adjusted P value [P-adj] = 8.9 × 10^−5^) and was less expressed in clusters 0 and 1 (Fig. 4 C and Fig. S2 C), indicating its transient upregulation during positive selection. We obtained very similar results with another scRNA-seq dataset (Duan et al., 2021), which showed the highest *Prmt1* expression in the Myc^+^ cell cluster and in which the subclustering of Myc^+^ cells (*n* = 324) yielded three subclusters, with the highest *Prmt1* expression coinciding with Myc and mTORC activation, rather than G2/M or post-GC signatures (Fig. S2 D).

To better establish the timing of *Prmt1* upregulation in the GC cycle, we took advantage of a scRNA-seq dataset of GCBC sorted at different times after positive selection (Pae et al., 2021). In this experiment, adoptively transferred B cells *Ly75*^*+/+*^ (encoding the surface protein DEC205) were specifically provided with antigen (conjugated to an anti-DEC205 antibody), which synchronizes their progression from the LZ into the DZ. We found that *Prmt1* expression was maximal 12 h after antigen delivery, coinciding with Myc and mTOR signatures peaks, and progressively declined from 30 h onwards (Fig. 4 D). Since *Ly75*^*+/+*^ B cells are synchronized at positive selection 12 h after antigen delivery and then enter the DZ and activate the E2F signature at 30 h and later times (Pae et al., 2021), our analysis placed Prmt1 upregulation at the positive selection. Accordingly, the unselected control *Ly75*^*−/−*^ B cells, which do not uptake the antigen, showed the lowest Prmt1 expression (Fig. 4 D).

We conclude that, while *Prmt1* is ubiquitously expressed in GCBC, it is transiently upregulated in positively selected GCBC, suggesting a need for higher Prmt1 activity at this point.

### Myc and mTORC1 regulate *Prmt1* expression

We probed the mechanism responsible for increasing *Prmt1* expression in positively selected cells. Myc was a likely candidate given the overlap of Prmt1 expression with other upregulated Myc targets (Fig. 4 C and Fig. S2 D). We found two candidate Myc binding sites (E-boxes) within the first intron of *Prmt1*, which coincided with the region occupied by *Myc* in activated B cells (Fig. 4 E; ChIP-seq dataset from Chou et al., 2016). These data implicated Myc in the upregulation of *Prmt1* in B cells.

Given that *Prmt1* upregulation in GCBC also coincided with mTORC1 activation (Fig. 4, C and D; and Fig. S2 D), we tested if mTORC1 activity was required for optimal *Prmt1* expression. Indeed, treating activated B cells with rapamycin caused a dose-dependent reduction in Prmt1 transcript and protein (Fig. 4 F). Moreover, gene expression data from mouse GCBC that had received antigen via the anti-DEC205 system (Ersching et al., 2017) showed that rapamycin prevented *Prmt1* upregulation during positive selection in vivo (Fig. 4 G).

We conclude that *Prmt1* is specifically upregulated in GCBC via Myc and mTORC1, coinciding with positive selection.

### Prmt1 promotes GCBC LZ to DZ transition

**Figure 5.**
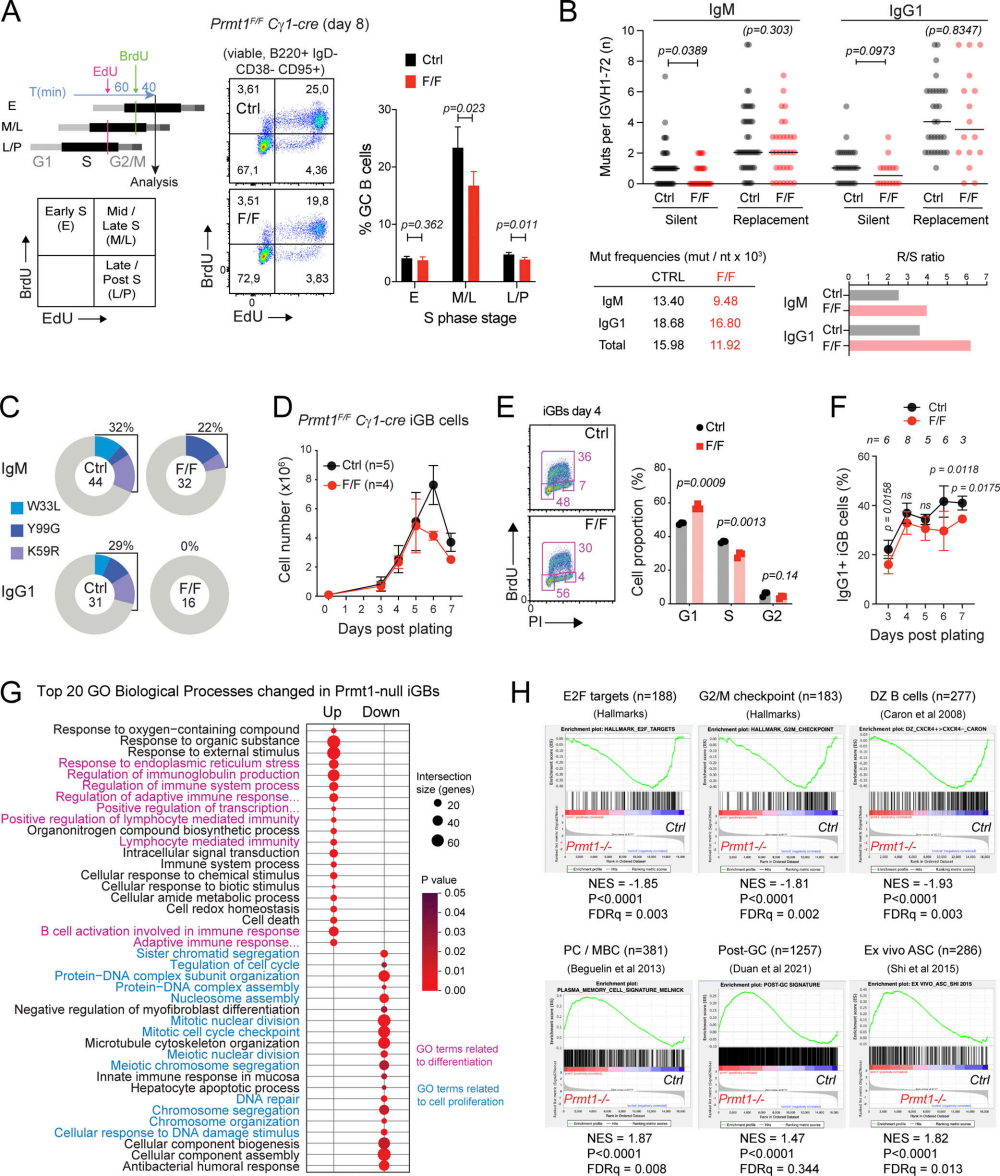
**PRMT1 is needed for GCBC transition from LZ to DZ. (A)** Scheme of the EdU/BrdU double-pulse cell labeling assay to assess S-phase progression. Representative flow cytometry plots distinguishing EdU and BrdU incorporation in GCBC and mean proportions + SD of four mice from two experiments. P values by Student’s *t* test are indicated in the figure. **(B)** SHM in GCBC sorted from NP-CGG immunized mice. Clockwise: Silent (S) and replacement (R) mutations in individual IgM and IgG using IGVH1-72 within a unique VDJ rearrangement (symbols), with medians (lines). P values calculated by Mann–Whitney test. The table of mutation frequencies is indicated in the figure. The plot of calculated replacement to substitution ratios. **(C)** Pie charts of the proportion of n sequences (indicated at center) with each affinity-enhancing mutation. **(B and C)** Data compiled from two experiments with two to three mice pools per genotype each. **(D)** Growth curve of iGBs derived from Cγ1-cre (Ctrl) or *Prmt1*^*F/F*^ Cγ1-cre (F/F) mouse splenic B cells plated on 40LB feeder cells (+1 ng/ml IL-4). Mean + SEM from two experiments. **(E)** Cell cycle profile of Cγ1-cre (Ctrl) and *Prmt1*^*F/F*^ Cγ1-cre (F/F) iGBs pulsed with BrdU (10 μM) for 1 h at 4 d after plating. Representative staining with anti-BrdU and propidium iodide (PI) and mean + SD for three mice per genotype from two experiments. **(F)** Mean ± SD proportion of IgG1^+^ iGB cells for *n* mice from five experiments as in D. **(G)** Functional annotation of genes differentially expressed *Prmt1*^*F/F*^ Cγ1-cre versus Cγ1-cre control iGBs. Differentially expressed changes (P-adj < 0.05) were ranked by expression level (base mean), and genes up- (318) and down- (388) regulated with base mean >100 were analyzed separately with gProfiler. **(H)** Selected transcriptional signatures analyzed by GSEA.

Positive selection of GCBC promotes their progression through the S-phase of the cell cycle, which starts in the LZ but is completed mostly within the DZ (Gitlin et al., 2014; Gitlin et al., 2015). We thus examined S-phase progression as a proxy for determining if Prmt1 deficiency compromised LZ to DZ entry. We sequentially pulsed immunized mice with the nucleoside analogs EdU and BrdU (Gitlin et al., 2014; Gitlin et al., 2015) and measured the proportion of GCBC in different S-phase stages according to their single or double nucleotide incorporation (Fig. 5 A). While Prmt1-null and control GCBC were similarly distributed in the early S-phase, there was a significant reduction in the proportion of Prmt1-deficient GCBC reaching the mid/late-S and late/post-S phases (Fig. 5 A).

We also analyzed SHM, which accrues with each round of cell division in the DZ (Gitlin et al., 2014). We sorted GCBC from mice immunized with NP-CGG at the peak of the GC reaction and analyzed SHM in IgM and IgG1 VDJ rearrangements using VH186.2 (IGHV1-72), which is preferentially selected by NP (Cumano and Rajewsky, 1985). The overall SHM accumulation at IGHV1-72 was lower in the Prmt1-null than control, with IgM showing a larger decrease than IgG1 (Fig. 5 B). Discriminating silent (S) and replacement (R) mutations further showed a significant reduction in the frequency of S mutations in IgM and a clear trend in IgG1 (Fig. 5 B). Accordingly, the VH186.2 sequences from Prmt1-null GCBC displayed a higher R/S mutations ratio than controls (Fig. 5 B). We also found that the well-described mutations W33L, Y99G, and K59R, which are each sufficient to confer high affinity against NP (Allen et al., 1988; Furukawa et al., 1999), were much less frequent in the Prmt1-null GCBC than controls (Fig. 5 C). We interpret these data as indicating that SHM accumulation is impaired, as shown by the frequency of the mutations that are not phenotypically selected (silent), which would thus reduce the probability of acquiring the W33L, Y99G, or K59R mutations. Supporting this, replacement mutations were less focused on hotspots in Prmt1-null compared with WT IgV sequences (Fig. S2 E). On the other hand, the increased R/S ratio suggests ongoing affinity selection of Prmt1-null B cells in the GC of *Prmt1*^*F/F*^ Cγ1-cre mice.

Together, the defects in S-phase progression and SHM accumulation are consistent with Prmt1-null undergoing fewer DZ cycles than control GCBC.

### Prmt1 regulates B cell proliferation after activation

**Figure S3.**
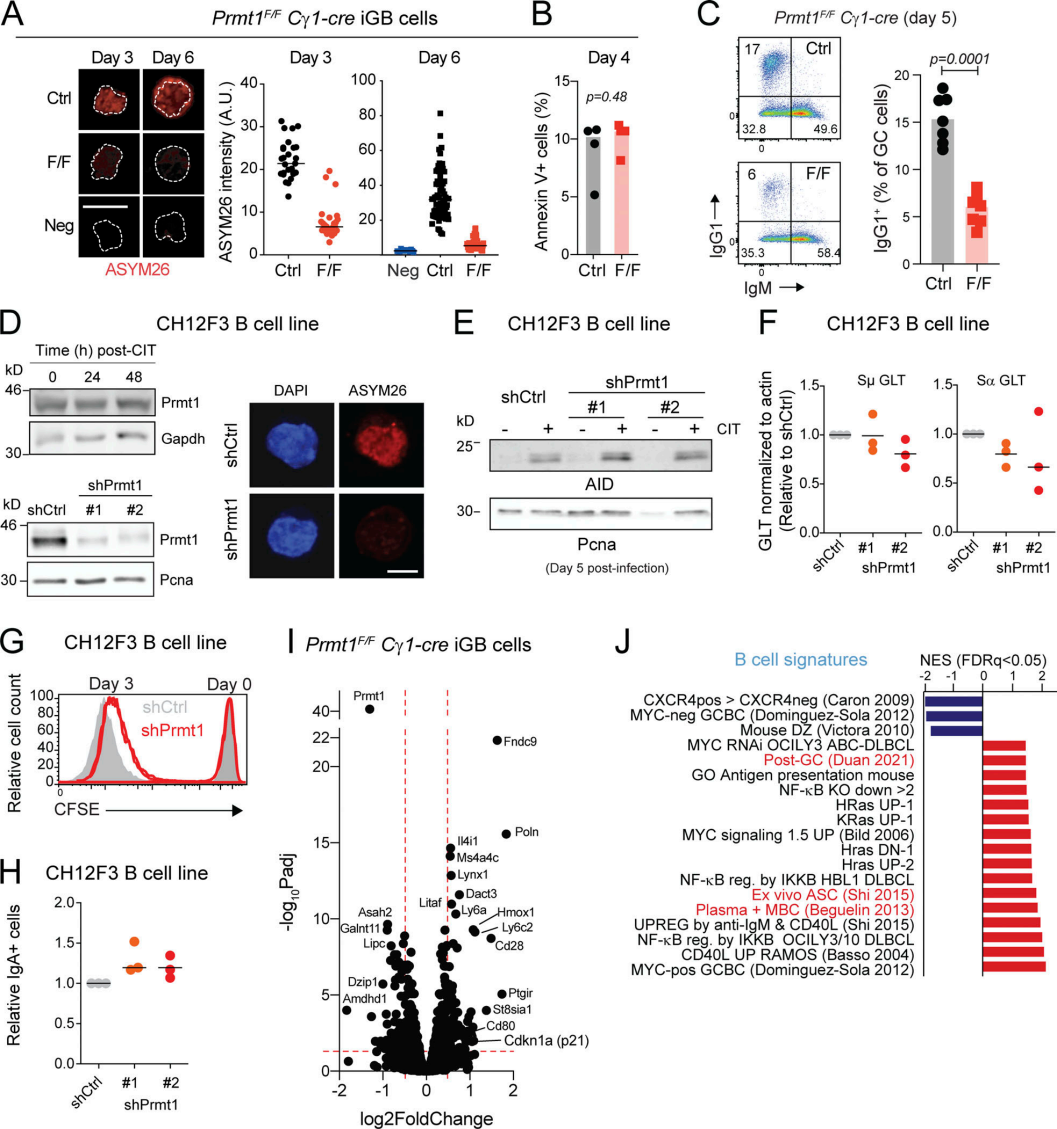
**Prmt1 depletion in iGBs and CH12F3 B cells.** Related to Fig. 5. **(A)** Overall aDMA levels estimated by IF with ASYM26 antibody in iGBs derived from Cγ1-cre (Ctrl) or *Prmt1*^*F/F*^ Cγ1-cre (F/F) mouse splenic B cells on days 3 and 6 after plating. Nuclei (dotted lines) were traced based on DAPI staining. Total signal was quantified and plotted for individual cells (symbols) with means (lines). Scale bar, 10 µm. **(B)** Mean ± SD proportion of Annexin-V^+^ Cγ1-cre (Ctrl) and *Prmt1*^*F/F*^ Cγ1-cre (F/F) cells in iGBs cultures at day 4. Individual mice (symbols) and means (lines) from two experiments are shown. **(C)** Representative flow cytometry and frequency of IgG1^+^ GCBC (B220^+^ IgD^−^ GL7^+^ CD95^+^) in *Prmt1*^*F/F*^ Cγ1-cre (FF) and Cγ1-cre (Ctrl) mice, 5 d after immunization, from three experiments. **(D)** Counterclockwise: Prmt1 and Gapdh (loading control) probed by Western blot in CH12F3 B cells over times post-CIT (1 μg/ml anti-CD40, 10 ng/ml IL-4, 1 ng/ml TGFβ). Western blot of Prmt1 and Pcna (loading control) in CH12F3 cells expressing control shRNA or two different shRNAs (#1 and #2) targeting Prmt1. Representative IF with ASYM26 showing efficient depleting of aDMA in CH12F3 cells expressing shRNA control or against Prmt1. Scale bar, 10 µm. **(E)** AID and Pcna (loading control) by Western blot in CH12F3 in cells treated as in D. **(F)** Sµ and Sα germline transcripts (GLT) measured by RT-qPCR in CIT-stimulated CH12F3 cells expressing shRNAs as in D), from three experiments. **(G)** CFSE histogram of CH12F3 B cells expressing shRNAs just after staining (day 0) and 3 d after CIT stimulation. Representative of two experiments. **(H)** Proportion of IgA^+^ in CH12F3 cells expressing shRNAs as in D at day 3 after CIT from three independent experiments (dots) normalized to shCtrl cells. Lines indicate means. **(I)** Volcano plot of transcriptional changes in *Prmt1*^*F/F*^ Cγ1-cre versus Cγ1-cre iGBs at day 3.5, by RNA-seq (three biological replicates per genotype). Dashed lines indicate thresholds (>1.4-fold change in either sense, P-adj < 0.05). **(J)** Transcriptional signatures significantly down (blue bars) or up (red bars) in Prmt1-null iGBs, ranked by normalized enrichment score (NES) calculated by GSEA for an ad hoc signature collection (see Table S1). **(B and C)** P values by unpaired, two tailed Student’s *t* test are indicated in the figure. Source data are available for this figure: SourceData FS3.

To uncover B cell–intrinsic effects of Prmt1, we expanded *Prmt1*^*F/F*^ Cγ1-cre naive B cells ex vivo by co-culturing with feeder cells that provided CD40L and BAFF. In this system, B cells acquire a GC-like phenotype (iGBs) and switch to IgG1 (Nojima et al., 2011; Litzler et al., 2019). *Prmt1*^*F/F*^ Cγ1-cre iGBs initially grew similarly to the control but then slowed down (Fig. 5 D). This was not due to inefficient Prmt1 excision, as reduced aDMA was evident by day 3 without any noticeable outgrowth of unexcised cells (Fig. S3 A). *Prmt1*^*F/F*^ Cγ1-cre iGBs had similar apoptosis levels to the controls (Fig. S3 B) but showed cell cycle arrest in G1 (Fig. 5 E), consistent with reduced proliferation. Switching to IgG1 in the *Prmt1*^*F/F*^ Cγ1-cre iGBs was largely normal, being slightly lower at the time points when proliferation was reduced (Fig. 5 F). *Prmt1*^*F/F*^ Cγ1-cre mice showed a lower proportion of IgG1^+^ GCBC compared with controls (Fig. S3 C), but since the probability of isotype switching increases with each cell division after B cell activation (Hasbold et al., 1998), we ascribed the switching deficit in vivo to the reduced proliferation of the Prmt1-null B cells. To rule out any requirement of Prmt1 in isotype switching, we used the CH12F3 B cell line, which switches from IgM to IgA upon cytokine stimulation (Nakamura et al., 1996). CH12F3 cells constitutively expressed Prmt1, which was efficiently reduced by shRNA (Fig. S3 D). Prmt1 deficiency did not significantly affect AID expression or germline switch transcripts levels (Fig. S3, E and F). Like in primary B cells, Prmt1 depletion reduced CH12F3 B cell proliferation (Fig. S3 G), but switching to IgA was normal (Fig. S3 H).

**Figure S4.**
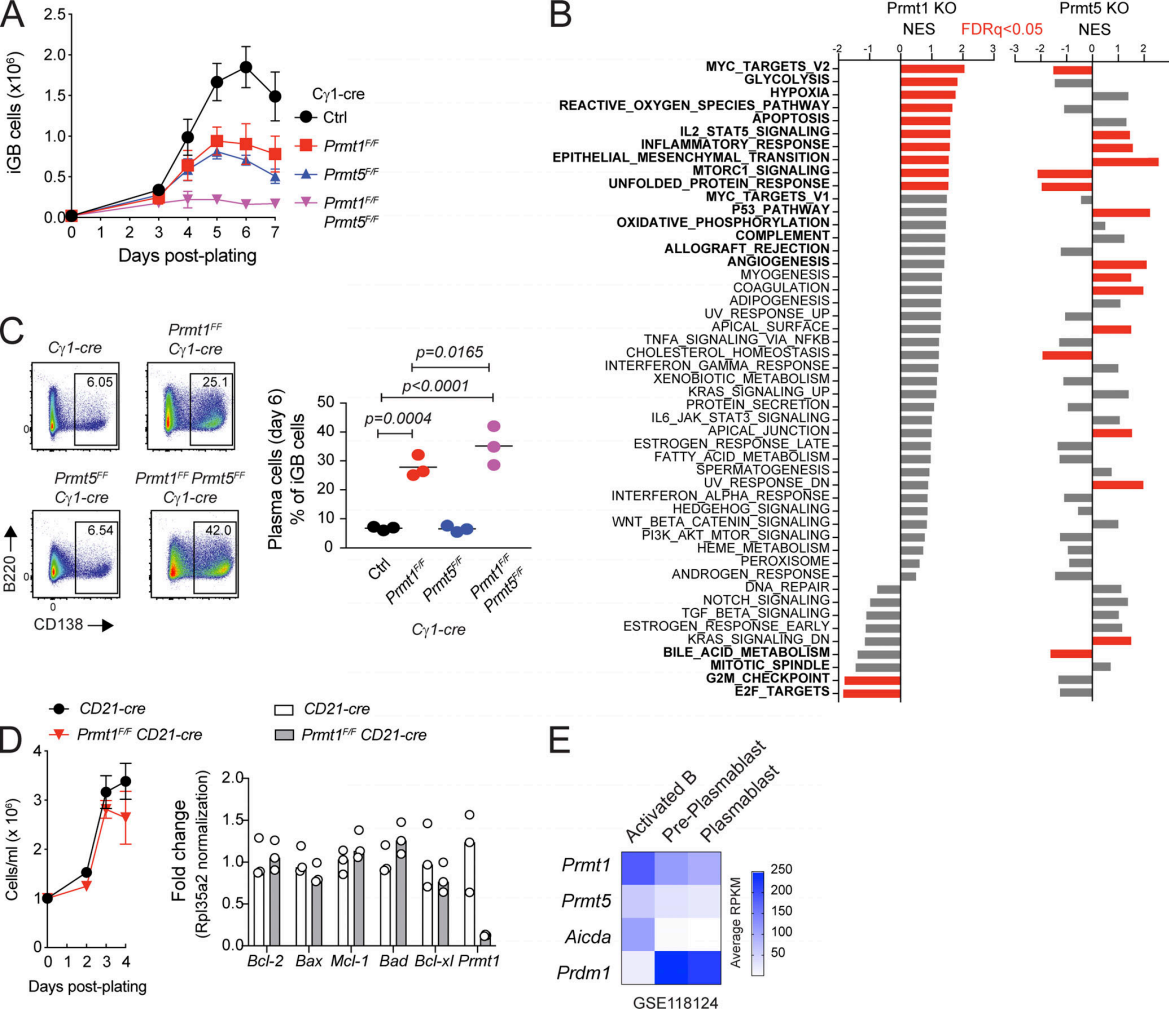
**Non-redundant function of PRMT1 and PRMT5 in B cells.** Related to Figs. 5 and 7. **(A)** Mean + SD cell number in iGB cultures derived from splenic B cells from control, *Prmt1*^*F/F*^, *Prmt5*^*F/F*^, or *Prmt1*^*F/F*^ *Prmt5*^*F/F*^, all in Cγ1-cre background, mice. Three mice/genotype from two experiments. **(B)** RNA-seq data obtained from iGBs from *Prmt1*^*F/F*^ Cγ1-cre versus Cγ1-cre, and a similar experiment comparing *Prmt5*^*F/F*^ Cγ1-cre versus Cγ1-cre iGBs (Litzler et al., 2019) were analyzed in parallel by GSEA against the Hallmark gene signature collection. **(C)** The proportion of PC in the cultures from A, determined by CD138 staining by flow cytometry. Significant P values by one-way ANOVA, except Prmt1 vs. double ko by unpaired two-tailed Student’s *t* test, are indicated in the figure. **(D)** Enumeration of cell number after activation with LPS + IL-4 of purified splenic B cells from CD21-cre *Prmt1*^*F/F*^ or CD21-cre controls (three mice/point from two experiments). Expression of the indicated pro- and anti-apoptotic genes in cDNA obtained 48 h after activation by RT-qPCR. **(E)** Expression of the indicated genes as average RPKM from two samples (GSE11812; Andreani et al., 2018).

To identify biological processes intrinsically regulated by Prmt1 in B cells, we generated RNA-seq data of WT and Prmt1-null iGBs. There were 706 differentially expressed genes (318 up- and 388 downregulated) in *Prmt1*^*F/F*^ Cγ1-cre versus control iGBs at day 4 after plating (Fig. S3 I). Consistent with the alterations observed in vivo and ex vivo, PRMT1-null B cells downregulated gene signatures related to cell proliferation and DZ GCBC (Fig. 5, G and H; Fig. S3 J, and Table S1). We have shown that Prmt5 is also required for iGBs and GCBC proliferation (Litzler et al., 2019). Like *Prmt1*, *Prmt5* is another Myc target (Koh et al., 2015) and was upregulated in positively selected GCBC clusters (Fig. 4, B and C). However, iGBs lacking both *Prmt1* and *Prmt5* showed a larger proliferation defect compared with either one of the single deficiencies (Fig. S4 A), indicating they contributed independently to B cell proliferation. Accordingly, there was little overlap between the transcriptional signatures affected by each deficiency in iGBs (Fig. S4 B), showing that each enzyme regulated distinct biological processes in B cells.

We conclude that Prmt1 is dispensable for AID activity and isotype switching but supports activated B cell proliferation programs independently of Prmt5.

### PRMT1 limits MBC differentiation

**Figure 6.**
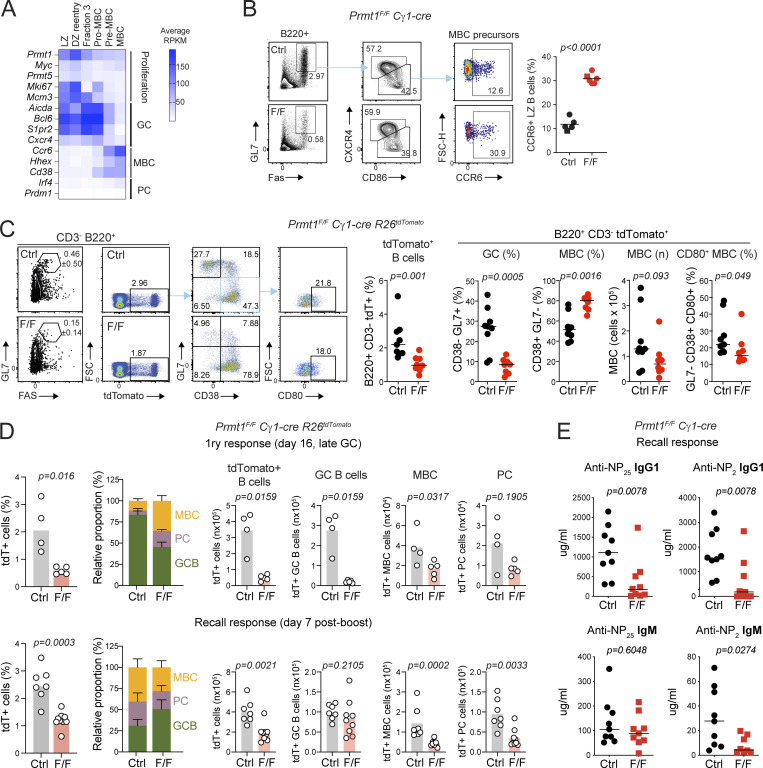
**PRMT1 limits MBC formation. (A)** Gene expression heatmap for *Prmt1* and selected markers. Average of three RNA-seq samples (GSE147095). **(B)** Representative flow cytometry and frequency of GC MBC precursors (CCR6^+^ LZ B cells) in individual *Prmt1*^*F/F*^ Cγ1-cre (F/F) and Cγ1-cre (Ctrl) mice, at 7–8 d after NP-CGG immunization, from two experiments. **(C)** Representative flow cytometry and frequency or absolute numbers of the indicated B cell subsets in *Prmt1*^*F/F*^ Cγ1-cre *R26*^*tdTomato*^ (F/F) and Cγ1-cre *R26*^*tdT*^ (Ctrl) mice 30 d after SRBC immunization, from four experiments. **(D)** Compiled frequency and absolute number of tdTomato (tdT)^+^ cells and their indicated subsets in *Prmt1*^*F/F*^ Cγ1-cre *R26*^*tdT*^ (F/F) and Cγ1-cre *R26*^*tdT*^ (Ctrl) mice 16 d after SRBC immunization and 7 d after SRBC recall, compiled from two experiments each. **(E)** Total (NP25) and high-affinity (NP2) serum NP-specific IgG1 and IgM titers were determined by isotype-specific ELISAs in mice immunized and recalled with NP-CGG. **(B–E)** Values for individual mice (symbols) and lines or bars indicating means (in B–D and F) or medians (in E). Significant P values (α = 0.05) by unpaired two-tailed *t* test (B–D, and F) or two-tailed Mann–Whitney test (E) are indicated in the figure.

The gene expression signatures found upregulated in Prmt1-null versus control iGBs were dominated by processes related to PC and MBC differentiation (Fig. 5, G and H; Fig. S3 J, and Table S1). Given that Prmt1 expression was inversely correlated with MBC differentiation (Fig. 6 A) and the increased proportion of B cells resembling MBC in *Prmt1*^*F/F*^ Cγ1-cre mice (Fig. 3, H and I), we analyzed this further. Indeed, immunized *Prmt1*^*F/F*^ Cγ1-cre mice showed a larger proportion of Ccr6^+^ MBC precursors in the GC LZ (Fig. 6 B). To further confirm MBC identity, we analyzed *Prmt1*^*F/F*^ Cγ1-cre *R26*^*tdTomato*^ mice 30 d after immunization. As expected, *Prmt1*^*F/F*^ Cγ1-cre *R26*^*tdTomato*^ mice generated less tdTomato^+^ cells than the Cγ1-cre *R26*^*tdTomato*^ controls (Fig. 6 C). However, the relative composition of these cells was different, with controls showing a larger proportion of GCBC (tdTomato^+^ CD38^−^ GL7^+^) than *Prmt1*^*F/F*^ Cγ1-cre *R26*^*tdTomato*^, which showed a larger proportion of MBC (tdTomato^+^ CD38^+^ GL7^−^), albeit the proportion of CD80^+^ MBC, which are thought to indicate GC origin (Shlomchik, 2017; Koike et al., 2019; Viant et al., 2021), was slightly reduced (Fig. 6 C). The absolute number of MBC produced was somewhat reduced between control and *Prmt1*^*F/F*^ Cγ1-cre *R26*^*tdTomato*^ mice, in line with the reduced number of tdTomato^+^ cells (Fig. 6 C).

To assess the quality of the Prmt1-null MBC, we compared the proportion and number of tdTomato^+^ GCBC, MBC, and PC cells between the primary and recall responses using the *Prmt1*^*F/F*^ Cγ1-cre *R26*^*tdTomato*^ mice. This experiment confirmed the relative increase in MBC generation from Prmt1-null B cells in the primary response (Fig. 6 D). It additionally revealed that these MBC failed to proportionally expand and/or generate PC at recall. Thus, as expected, the control mice showed a larger proportion of tdTomato^+^ PC and MBC at recall than at the primary response (Fig. 6 D), reflecting the expansion and differentiation of high-affinity MBC cells generated during the latter, as previously characterized for NP-CGG (Mesin et al., 2020). In contrast, despite *Prmt1*^*F/F*^ Cγ1-cre *R26*^*tdTomato*^ mice generating a larger proportion of MBC than controls in the primary response, they did not produce a proportionally larger MBC or PC expansion upon recall (Fig. 6 D). This result strongly suggested that the Prmt1-null MBC failed to expand because of poor affinity, consistent with the low anti-NP IgG1 titers and affinity of *Prmt1*^*F/F*^ Cγ1-cre (Fig. 2, A and B; and Fig. 6 E). To avoid any potential confounder from the reduced generation of switched B cells in vivo in *Prmt1*^*F/F*^ Cγ1-cre mice, we measured the anti-NP IgM response at recall. While total anti-NP IgM, which likely has a component of extra-follicular PC, was similar in both mice, *Prmt1*^*F/F*^ Cγ1-cre failed to produce the high-affinity anti-NP_2_ IgM that would originate from GC-experienced MBC at recall (Fig. 6 E).

Collectively, these results show that PRMT1 limits the generation of MBC in vivo but is required for the generation of high-affinity MBC.

### PRMT1 intrinsically limits PC differentiation

**Figure 7.**
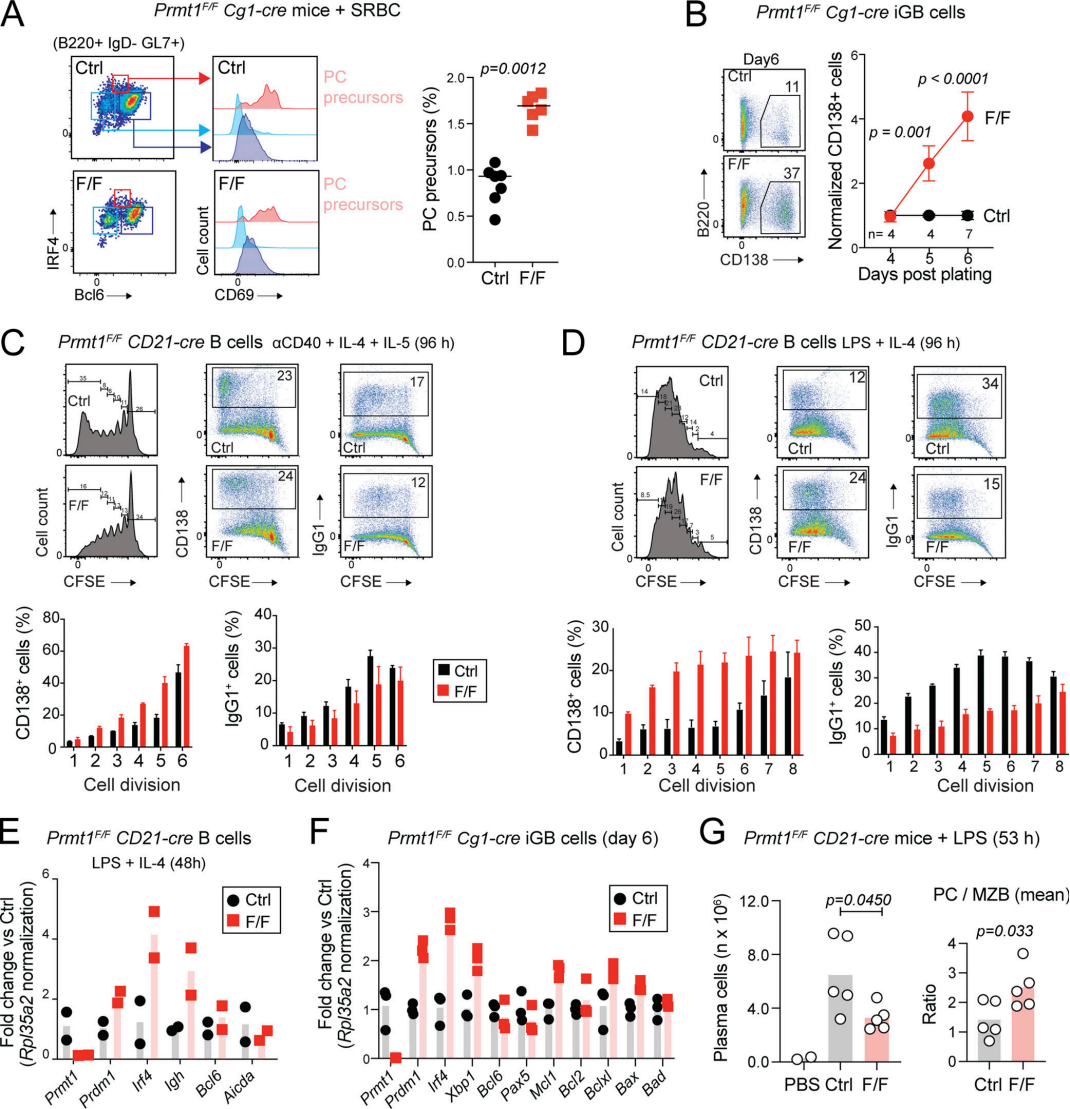
**PRMT1 limits PC differentiation. (A)** Representative flow cytometry to identify PC precursors (Bcl6^LOW^ IRF4^+^ CD69^+^) GCBC in *Prmt1*^*F/F*^ Cγ1-cre (F/F) and Cγ1-cre (Ctrl) mice. Values for individual mice (symbols) and means (lines) from two experiments. **(B)** Representative flow cytometry of CD138^+^ PC proportion in *Prmt1*^*F/F*^ Cγ1-cre (F/F) or Cγ1-cre (Ctrl) iGBs at day 6. Mean ± SD (normalized to Ctrl at day 4) of three experiments. **(C)** Purified CD21-cre (Ctrl) and *Prmt1*^*F/*F^ CD21-cre (F/F) splenic B cells were stained with CFSE and activated with agonist anti-CD40 (10 μg/ml), IL-4 (10 ng/ml), and IL-5 (5 ng/ml) for 96 h. Flow cytometry plots and mean + SEM proportion of CD138^+^ and IgG1^+^ cells per cell division for two mice per genotype from one representative of two experiments. **(D)** As in C, but activated with LPS (25 μg/ml) and IL-4. Representative of two experiments. **(E and F)** Transcript levels in individual mice (symbols) and means (bars) by RT-qPCR from cells in D and B, respectively. **(G)** PC (IgD^−^ CD3^−^ CD138^+^) in age-matched *Prmt1*^*F/F*^ CD21-cre (F/F) and CD21-cre (Ctrl) mice that had received intraperitoneal LPS 53 h prior. Left: Absolute PC number for each mouse (symbols) with means (bars), from three experiments. Right: Same values normalized to the mean MZB cell numbers in unimmunized mice of each genotype (Fig. 1 F). **(A, B, and G)** P values by unpaired, two-tailed Student’s *t* test are indicated in the figure.

The tdTomato labeling experiments showed an increased proportion of PC in *Prmt1*^*F/F*^ Cγ1-cre *R26*^*tdTomato*^ than control mice after primary immunization, albeit with reduced absolute numbers (Fig. 6 D), likely due to the GC defect. For additional evidence of increased PC differentiation in vivo, we stained for a previously described population of GCBC that contains PC precursors (Ise et al., 2018). In addition to the larger Bcl6^−^ MBC population, the *Prmt1*^*F/F*^ Cγ1-cre mice showed a larger proportion of B220^+^ IgD^−^ GL7^+^ Bcl6^LOW^ Irf4^+^ CD69^+^ PC precursors (Fig. 7 A).

We thus tested if Prmt1 intrinsically regulated PC differentiation, which can be efficiently induced ex vivo. Indeed, the proportion of *Prmt1*^*F/F*^ Cγ1-cre iGBs expressing the PC marker CD138^+^ was about fourfold higher on day 6 after plating (Fig. 7 B). Moreover, Prmt1 deficiency had a much larger and independent intrinsic effect than Prmt5 for limiting PC differentiation when tested side by side (Fig. S4 C). Similar to isotype switching, the chances of PC differentiation increase with cell division numbers (Hasbold et al., 2004; Nutt et al., 2015). To distinguish whether Prmt1 loss increased the proportion of cells that eventually differentiated or accelerated the differentiation of individual cells, we measured PC differentiation per cell division. We loaded CD21-cre *Prmt1*^*F/F*^ mouse splenic B cells with CFSE before stimulating them with either anti-CD40 or LPS + IL-4. In either condition, Prmt1-null B cells generated a larger proportion of CD138^+^ from the earliest cell divisions (Fig. 7, C and D). Consistent with a differentiation process, isotype switching, which stops in plasmablasts (Hasbold et al., 2004), was concomitantly reduced (Fig. 7, C and D). Bona fide PC differentiation of the Prmt1-null B cells was confirmed by the upregulation of transcription factors *Irf4*, *Prdm1*, and *Xbp1*, as well as *Igh* and *Mcl1*, without any indication of pro-apoptotic gene changes (Fig. 7, E and F; and Fig. S4 D). Increased PC differentiation upon Prmt1 loss or inhibition was also consistent with Prmt1 downregulation during differentiation of ex vivo activated B cells (Fig. S4 E). For additional evidence of PC differentiation in vivo, we injected *Prmt1*^*F/F*^ CD21-cre mice with LPS, which generates PC mostly from MZB cells in a T cell–independent manner (Genestier et al., 2007), thus bypassing any GC defects. While *Prmt1*^*F/F*^ CD21-cre mice produced approximately twofold less PC than controls, when normalized by their threefold defect in MZB cells (Fig. 1 F), there was a significant increase in the relative proportion of PC differentiation (Fig. 7 G). We conclude that Prmt1 intrinsically limits PC differentiation after activation.

### PRMT1 prevents BCL cell differentiation

**Figure 8.**
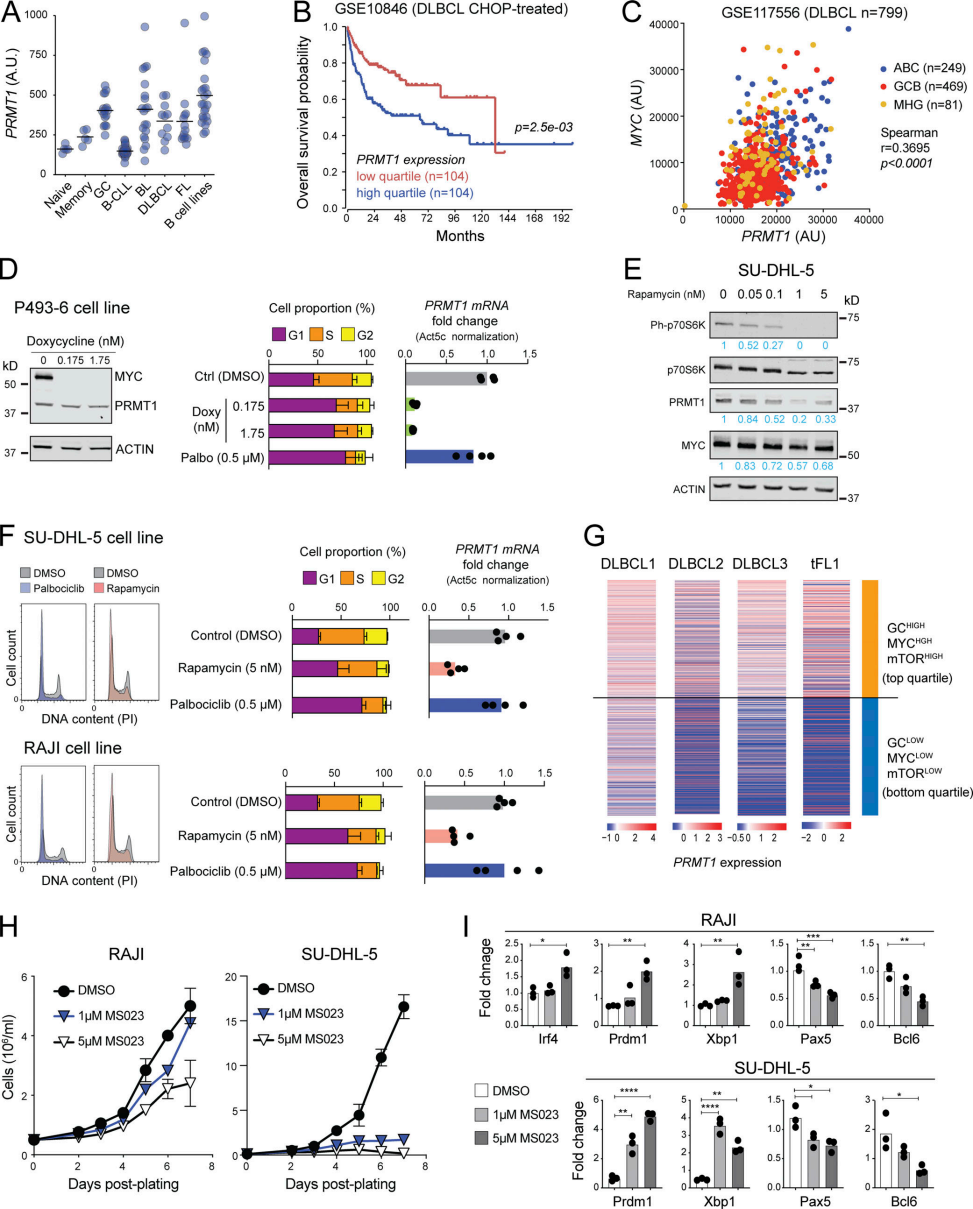
**PRMT1 prevents BCL differentiation. (A)** PRMT1 expression (microarray dataset GSE2350) in human B cell subsets, samples of B cell chronic lymphocytic leukemia (B-CLL), BL, DLBCL, follicular lymphoma (FL), and a collection of BCL cell lines. **(B)** Kaplan–Meier survival plot of DLBCL patients, stratified by PRMT1 expression. **(C)** Correlation between *MYC* and *PRMT1* expression in DLBCL samples indicating subtype (ABC, activated B cell like; GCB, GCBC like; MHG, molecular high grade). **(D)** Western blot for MYC and PRMT1 in the human B lymphoblastoid line P493-6 treated with vehicle or doxycycline for 24 h to repress MYC expression. Bar plots show mean + SD of cell cycle stages distribution and *PRMT1* transcript levels in each condition for four biological replicates from two experiments in cells treated with doxycycline or the CDK4/6 inhibitor palbociclib. **(E)** Western blot probing the mTORC1 target P70S6k and indicated proteins in whole SU-DHL-5 cell extracts treated with rapamycin for 24 h. **(F)** Cell cycle distribution of SU-DHL-5 and RAJI cells treated with palbociclib, or mTORC inhibitor rapamycin. Bar plots show mean + SD of cell cycle stages distribution and PRMT1 transcript levels in each condition for four biological replicates from two experiments. **(G)** Heatmap of *PRMT1* expression in single cells from human DLBCL and tFL, comparing the top and bottom quartiles of each sample ranked according to the enrichment in a combined (GC + MYC targets + mTORC1 targets) gene expression signature. **(H)** Cell proliferation of RAJI and SU-DHL-5 cells in the presence of type I PRMT inhibitor MS023. **(I)** Relative expression of the indicated genes measured by RT-qPCR in RAJI and SU-DHL-5 cells treated for 7 d with 1 or 5 µM MS023. Significant differences by one-way ANOVA with Tukey’s post-test (*, P < 0.05; **, P < 0.01; ***, P < 0.001; ****, P < 0.0001). Source data are available for this figure: SourceData F8.

**Figure S5.**
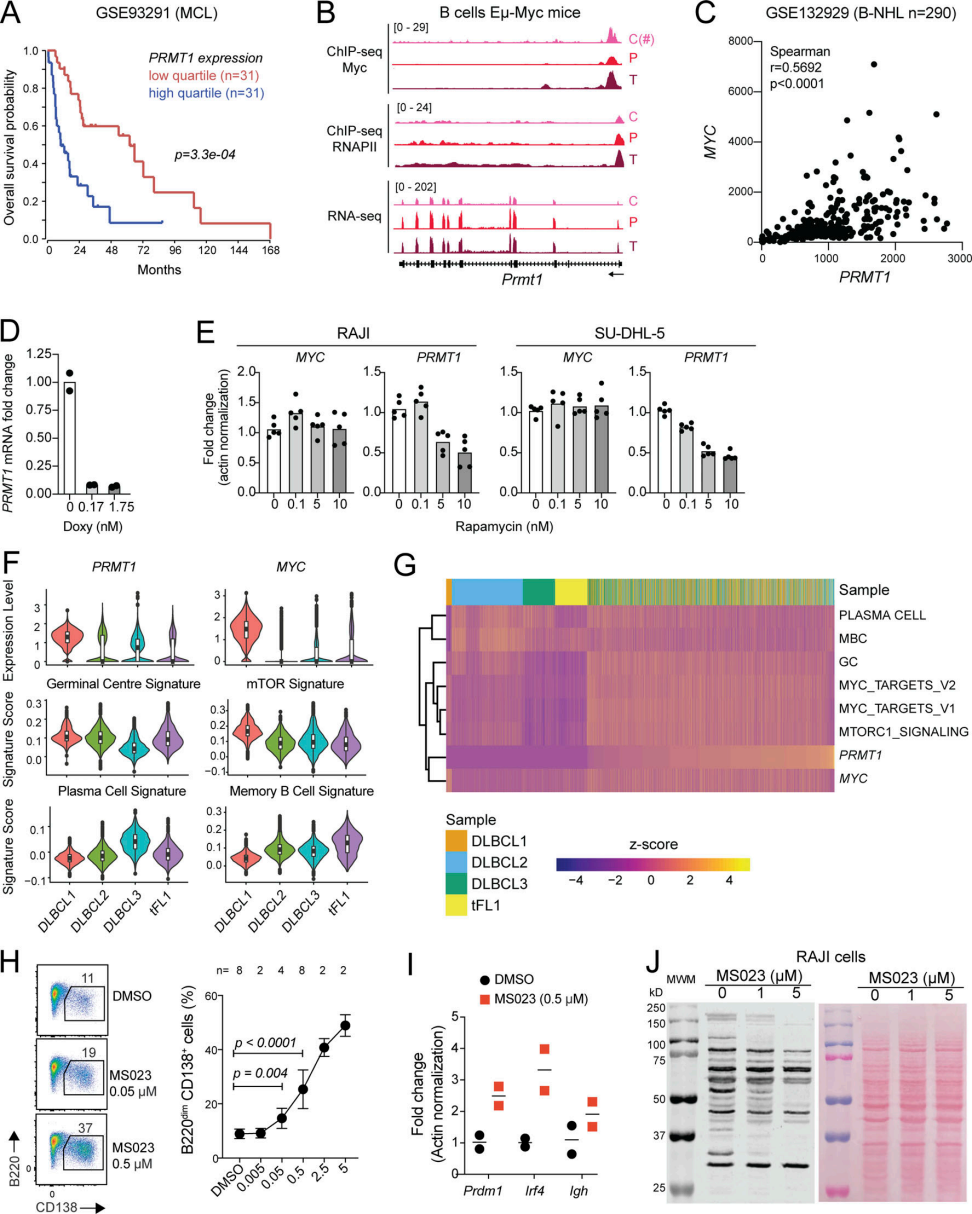
**PRMT1 expression in BCL.** Related to Fig. 8. **(A)** Kaplan–Meier survival plot of mantle cell lymphoma patients stratified by *PRMT1* expression. **(B)** ChIP-seq tracks (GSE51011 dataset) showing Myc and RNAPII occupancy at *Prmt1* in B220^+^ splenic B cells from WT (C) or Eµ-Myc mice in pretumor (P) or tumor (T) stage; and RNA-seq tracks of *Prmt1* expression in the same cells. (C#; this Myc ChIP-seq sample was done separately from the others and showed batch effects that compromise quantitative comparison to the P and T samples but confirms Myc occupation of *Prmt1* in normal B cells.) **(C)** Correlation between *MYC* and *PRMT1* expression for the indicated dataset of DLBCL samples (subtypes not discriminated). **(D)** *PRMT1* transcript levels by RT-qPCR in P493-6 cells after repressing *MYC* expression by doxycycline treatment. **(E)** *MYC* and *PRMT1* transcript levels by RT-qPCR in the RAJI and SU-DHL-5 cell lines treated with rapamycin. **(F)** Violin plot of overall *PRMT1* and *MYC* expression, as well as module scores for the indicated signatures in the four BCL from human patients analyzed in Fig. 8 G. **(G)** Heatmap of *PRMT1* and *MYC* expression, together with ssGSEA scores for selected signatures in individual cells from the samples in F after integration. Values were z-score normalized and clustered using Euclidian distances. Columns were sorted according to PRMT1 expression. **(H)** Representative flow cytometry and mean ± SEM PC proportion in cultures of naive B cells (from *n* mice) stimulated with LPS (5 µg/ml) + IL-4 (5 ng/ml) and treated with different doses of MS023 for 4 d, from two to four experiments. **(I)** Expression of the indicated genes determined by RT-qPCR in cells from two experiments from H). **(J)** Western blot of aDMA-modified proteins (ASYM26) in whole RAJI cells lysates treated as indicated for 7 d. Source data are available for this figure: SourceData FS5.

Given the effect of PRMT1 in preventing differentiation of activated B cells and since differentiation is a tumor suppressor phenomenon, we asked if PRMT1 played a similar role in BCL cells. PRMT1 was highly expressed in human BCL samples, roughly correlating with their proliferation: highest in Burkitt’s lymphoma (BL) and diffuse large BCL (DLBCL), and lowest in indolent chronic lymphocytic leukemia (Fig. 8 A). Moreover, high PRMT1 expression was associated with reduced survival in DLBCL and mantle cell lymphoma patients (Fig. 8 B and Fig. S5 A).

As in normal mouse B cells, Myc occupied *Prmt1* in the mouse BCL cells that develop in the Eµ-cMyc transgenic model, with Myc overexpression also leading to higher RNAPII occupancy (Fig. S5 B; Sabò et al., 2014). Accordingly, the expression of both genes was significantly correlated in human BCL samples (Fig. 8 C and Fig. S5 C), and repressing *MYC* in the BL-like P493-6 B cell lymphoblastoid cell (Schuhmacher et al., 1999) downregulated *PRMT1* transcription (Fig. 8 D and Fig. S5 D). The similarities in *PRMT1* expression regulation between normal mouse B cells and human BCL extended also to the mTORC1 requirement. Rapamycin treatment downregulated *PRMT1* expression in the BL cell line RAJI and the DLBCL cell line SU-DHL-5 (Fig. 8, E and F; and Fig. S5 E). *PRMT1* downregulation was not a consequence of the cell cycle arrest caused by MYC depletion or mTORC inhibition, as shown by the CDK4/6 inhibitor palbociblib that induced G1 arrest but not PRMT1 downregulation (Fig. 8, D and F). We then examined the expression of PRMT1 in scRNA-seq data from three DLBCL and one transformed follicular lymphoma (tFL) clinical samples (Roider et al., 2020). As expected, the samples differed in their relative overall expression of *PRMT1*, *MYC*, and other signatures (Fig. S5 F). However, at the single-cell level, PRMT1 expression was largely restricted to cells in which the GC, MYC, and mTORC1 signaling signatures were overall higher than PC and MBC signatures across samples (Fig. S5 G). Moreover, stratifying the cells by a combined (GC + MYC targets + mTORC1 signaling) score showed significantly higher PRMT1 expression in the top than the bottom quartile (Fig. 8 G), highlighting the conserved link between PRMT1 and MYC and mTORC1 activity.

There are no PRMT1-specific inhibitors, but inhibitors of type I PRMTs are in clinical trials and one has been shown to reduce the proliferation of human BCL cell lines in vitro and after xenotransplantation into mice (Fedoriw et al., 2019). A very similar inhibitor, MS023, mimicked the effect of Prmt1 ablation in mouse splenic B cells, increasing the differentiation to PC in a dose-dependent manner (Fig. S5, H and I). MS023 also reduced asymmetric protein arginine methylation and impaired the proliferation of RAJI and SU-DHL-5 BCL cell lines (Fig. 8 H and Fig. S5 J). Moreover, both BCL cell lines upregulated *BLIMP1*, *XBP1*, and *IRF4* and downregulated *PAX5* and *BCL6* when treated with MS023 (Fig. 8 I), indicative of PC differentiation.

We conclude that *PRMT1* is regulated in a MYC- and mTORC1-dependent manner in BCL cells to sustain lymphoma cell proliferation and also to prevent their differentiation.

## Discussion

We show that PRMT1 is a determinant of activated and GCBC fate that is necessary to promote antibody affinity maturation. Our data are consistent with previous reports showing that PRMT1, while necessary for B cell development, is dispensable for follicular B cells, yet required for MZB cell homeostasis and GC formation (Dolezal et al., 2017; Hata et al., 2016; Infantino et al., 2017). However, the mouse models used so far were not adequate for probing the functions of PRMT1 in GCBC. Thus, eliminating PRMT1 in B cell precursors can select compensatory mechanisms, as implied by the normal T-dependent antibody response in *Prmt1*^*F/F*^ CD19-cre mice (Hata et al., 2016). In contrast, Prmt1 ablation in resting B cells in *Prmt1*^*F/F*^ CD23-cre mice not only reduced antibody responses but also uncovered an activation defect with increased apoptosis upon stimulation of the Prmt1-depleted naive B cells, which compromises GC formation (Infantino et al., 2017). By using *Prmt1*^*F/F*^ Cγ1-cre mice, which delete *Prmt1* after B cell activation and bypassed activation defects, we show that Prmt1 enables GC expansion and pinpoints positive selection as a stage at which Prmt1 is especially required in GCBC.

The primary antibody response in *Prmt1*^*F*^ ^*/F*^ Cγ1-cre mice is compromised in titer and affinity. Reduced titer can be explained by fewer PC, likely a consequence of premature differentiation of PC extrafollicularly, thus limiting clonal expansion after B cell activation, as well as of smaller GC due to impaired expansion. The affinity defect is explained by the latter hampering affinity maturation. The GC defect can also explain why the recall antibody response of *Prmt1*^*F/F*^ Cγ1-cre mice is more drastically affected than the primary. The tdTomato lineage tracing experiments show that consistent with enhanced differentiation, *Prmt1*^*F/F*^ Cγ1-cre mice generate a larger proportion of MBC and PC in the primary response. However, the Prmt1-null MBC fail to expand upon recall, in contrast to the control mice. Together with the specific deficit in high-affinity anti-NP IgM upon recall, these observations imply that high-affinity MBC that dominate this type of recall response (Mesin et al., 2020) are scarce in the absence of Prmt1. It is possible that some Prmt1-null B cells differentiate to MBC prior to the GC and would thus have low affinity. However, several lines of evidence indicate a GC defect in *Prmt1*^*F/F*^ Cγ1-cre mice that additionally impairs the formation of high-affinity MBCs. First, antibody affinity was reduced to a greater extent for the anti-CGG response, which requires more interclonal competition, than for the clonally restricted anti-NP response (Finney et al., 2018; Bannard and Cyster, 2017). Second, the increased R/S ratio was caused by a reduced number of silent mutations and scarcity of the anti-NP affinity-enhancing W33L, Y99G, and K59R mutations (Allen et al., 1988; Furukawa et al., 1999). This suggests that the GC is selecting from the mutations available, but because SHM is less frequent, the W33L, Y99G, and K59R mutations are just less likely to happen. This scenario is consistent with the Prmt1-null B cells going through the DZ fewer times (Victora and Nussenzweig, 2022; Gitlin et al., 2014). Third, Prmt1-null GCBC initiate S-phase but display impaired progression to mid, late, and post S-phase, which take place in the DZ (Gitlin et al., 2014; Pae et al., 2021; Victora et al., 2010). Fourth, *Prmt1* upregulation upon positive selection by stimuli that are known to promote DZ reentry implies an important role at this stage. Thus, while *Prmt1* is expressed in most GCBC cells, it is substantially upregulated by a mechanism requiring Myc and mTORC1 signaling, which are activated in positively selected GCBC to prepare them for cell division and SHM in the DZ (Dominguez-Sola et al., 2012; Calado et al., 2012; Ersching et al., 2017; Finkin et al., 2019; Pae et al., 2021) *Myc* occupies *Prmt1* intronic elements in B cells and *Prmt1* is synchronously upregulated with the Myc targets signature in GCBC, suggesting a direct regulation. We propose that mTORC1 signaling contributes to the upregulation based on the coincident expression of *Prmt1* and mTORC1-regulated genes in GCBC and the repression of *Prmt1/PRMT1* upon mTORC1 inhibition. Future work will define whether mTORC1 activity upregulates *Prmt1* transcription via STAT1, as in human hepatic cells (Zhang et al., 2021), or by another mechanism. Since Prmt1 is expressed in all GCBC and protein arginine demethylation largely proceeds by substrate turnover (Xu and Richard, 2021), the need for *Prmt1* upregulation after positive selection might seem puzzling. However, it is conceivable that if a large proportion of its substrates were coupregulated during positive selection, such as the abundant Myc target genes, more PRMT1 would be necessary to ensure their quantitative modification. Given the multiplicity of PRMT1 substrates (Xu and Richard, 2021), it is unlikely that all the phenotypes we find can be explained by any single Prmt1 substrate.

Unlike in naive B cells (Infantino et al., 2017), PRMT1 is dispensable for survival once B cells have activated, but are required for proliferation. DZ B cell proliferation depends on E2F, enabled by cyclinD3 (Pae et al., 2021). A BTG2–PRMT1 complex has been shown to disrupt the interaction between cyclinD3 and CDK4 in pre-B cells, thus reducing proliferation in favor of differentiation (Dolezal et al., 2017). However, Prmt1-null mature B cells display less proliferation and more differentiation, the opposite of what it would be expected if Prmt1 acted as in pre-B cells. Instead, Prmt1-null iGBs upregulate *Cdkn1a* (p21) and accumulate in G1. Extrapolating from findings in other cell types, several non-mutually exclusive mechanisms could be at play, including direct modification or transcriptional regulation via histone methylation of cell cycle regulators (Xu and Richard, 2021), stimulating EZH2 activity (Li et al., 2021), or regulating Myc activity, as found in cancer cells (Favia et al., 2019; Tikhanovich et al., 2017; Hsu et al., 2021).

Prmt1 limits the differentiation in activated B cells, evidenced in *Prmt1*^*F/F*^ Cγ1-cre mice by the increase in the proportion of MBC and PC and their precursors. At least for PC, this is an intrinsic effect and can be GC-independent. The anti-differentiation effect of PRMT1 in mature B cells contrasts with its pro-differentiation function in pre-B (Dolezal et al., 2017), but is in line with the context-dependent function of PRMT1. Thus, PRMT1 limits the differentiation of epidermal progenitor cells (Bao et al., 2017) and megakaryocytes (Jin et al., 2018) but promotes oligodendrocyte (Hashimoto et al., 2016) and muscle stem cell differentiation (Blanc and Richard, 2017). The mechanism by which PRMT1 limits B cell differentiation is also likely to be multipronged. Increased MBC proportion in *Prmt1*^*F/F*^ Cγ1-cre mice is reminiscent of the phenotype caused by the loss of the Myc partner Miz1 (Toboso-Navasa et al., 2020). PRMT1 can methylate MYC in myeloid and glioblastoma cells and modulate its transcriptional activity (Favia et al., 2019; Tikhanovich et al., 2017). PRMT1 also modifies components of the BCR and NF-κB signaling pathways, whereby it could function upstream from Myc. Igα aDMA dampens pre-BCR signaling (Infantino et al., 2010), which seems conserved in mature B cells (Infantino et al., 2017). Thus, PRMT1 may increase the threshold for activating signaling pathways determining B cell fate (Luo et al., 2018; Shlomchik et al., 2019).

PRMT1 is highly expressed and likely prognostic in mature BCL, displaying MYC and mTORC dependency and similar functions as in activated B cells. Inhibiting type I PRMTs reduced human BCL cell proliferation, similar to PRMT1 depletion in the mouse BCL line CH12. The inhibitor also caused PC differentiation in BCL cells, mimicking the effect of inhibiting or deleting Prmt1 in mouse B cells. Differentiation was observed regardless of the relative sensitivity of BCL cell line proliferation to the inhibitor, which is explained by synergism with the de facto PRMT5 deficiency caused by MTAP deletion in SU-DHL-5 (Fedoriw et al., 2019). Accordingly, we show that PRMT1 and PRMT5 contribute independently to B cell proliferation and regulate distinct processes. Our results align with the synergistic effect of type I PRMT and PRMT5 inhibitors against BCL (Fedoriw et al., 2019; Fong et al., 2019) and improve the mechanistic insight into their consequences.

In conclusion, PRMT1 is an important regulator of B cell fate required for antibody responses, playing a special role after positive selection in the GC. Our findings suggest practical implications for the treatment of BCL and open new research avenues to identify the PRMT1 substrates that underlie its function in B cells.

## Materials and methods

### Mice

*Prmt1*^*f/f*^ mice (Yu et al., 2009) were backcrossed to C57BL6/J background for >10 generations and bred with Cγ1-cre mice (Casola et al., 2006), a kind gift from Dr. K. Rajewsky (Max Delbrück Center for Molecular Medicine, Berlin, Germany), or CD21-cre mice (Kraus et al., 2004) from Jackson Labs. Cre^+^ mice were always used as controls, backcrossed to the same background, and bred in parallel. *Aicda-GFP* mice (Crouch et al., 2007), a gift from Dr. R. Casellas (National Cancer Institute, Bethesda, MD, USA), and Rosa26^tdTomato^ (Jackson Labs) from Dr. W.-K. Suh (Institut de Recherches Cliniques de Montréal [IRCM], Montreal, Canada) were in C57BL6/J background (>10 generations). Age-matched groups of either males, females, or a balanced mix were used. No obvious sex-dependent differences were observed. Mice were kept at the IRCM-specific pathogens-free animal house. All work was reviewed and approved by the animal protection committee at the IRCM (protocols 2013-18, 2017-08, 2021-05).

### Immunization

Age- and sex-matched mice of 60–120 d of age were immunized either intraperitoneally with 50–100 μg NP_18_-CGG (Biosearch Technologies) in Imject Alum adjuvant (Thermo Fisher Scientific) or intravenously with 10^9^ SRBC in 200 μl PBS (IC100-0210; Innovative Research). Recall immunizations were done ∼18 wk after the primary immunization with 50 μg NP_18_-CGG or 10^6^ SRBC.

### Flow cytometry

Mononuclear cells from the mouse spleen were obtained by mashing through a 70-µm cell strainer with a syringe plunger. Cells suspensions were washed in PBS and resuspended in 1 ml of red blood cell lysis (155 mM NH_4_Cl, 10 mM KHCO_3_, 0.1 mM EDTA) for 5 min at room temperature and washed. Cells were resuspended in PBS 1% BSA, stained with combinations of antibodies listed in Table S2 for analysis of different lymphocyte populations, and passed through a 40-μm nylon cell strainer before acquisition. NP-specific cells were detected using NP_28_-PE (N-5070-1; Biosearch Technologies). Data were acquired using BD LSR Fortessa, BD Facscalibur (BD Biosciences), or SA3800 Spectral Analyzer (Sony Biotechnology) and analyzed using FlowJo (BD Biosciences). Sorting was done with a BD FACSARIA III (BD Biosciences) apparatus.

### EdU/BrdU staining to monitor S-phase progression

We followed the method described by Gitlin et al. (2015), with minor modifications. Mice immunized with SRBC 8 d prior were intravenously pulsed with 1 mg 5-ethynyl-2′-deoxyuridinae (EdU, Thermo Fisher Scientific Scientific) in 200 µl of PBS, followed 1 h later by an intraperitoneal injection of 2 mg 5-bromo-2′-deoxyuridinae (BrdU, Millipore Sigma) in 200 µl PBS. Mice were sacrificed 40 min after the BrdU pulse and 10^6^ splenocytes were stained for cell surface markers (anti-B220-AF700, IgD-APC, CD38-PE, and CD95-bio/SA-BV605) followed by an overnight cell fixation and permeabilization using the Cytofix/Cytoperm buffer (BD Pharmingen). Samples were then processed using the BD BrdU flow kit components (BD Pharmingen), except we used the anti-BrdU antibody clone MoBU-1 (Invitrogen) and the Click-iT PLUS EdU-Pacific Blue kit (Thermo Fisher Scientific) according to manufacturers’ instructions, except that we used half the amounts of all components of the click reaction. Samples were acquired on a BD Fortessa and analyzed using Flowjo. GCBC were gated as singlets/live/B220^+^/IgD^−^/Cd38^−^/Fas^+^. Early S-phase GCB cells were labeled as EdU-BrdU^+^, mid/late S-phase cells as EdU^+^BrdU^+^, and post-S phase cells as EdU^+^BrdU^−^.

### Primary B cell cultures

Naive primary B cells were purified from splenocytes by CD43^+^ cell depletion using anti-CD43 microbeads (cat. #130-049-801; Miltenyi), and an autoMACS (Miltenyi), or by using the EasySep Mouse B cell Isolation Kit (cat. #19854; Stem Cell) and the column-free magnet EasyEights (cat. #18103; Stem Cell), following manufacturer instructions. Primary B cells were cultured at 37°C with 5% (vol vol^−1^) CO_2_ in RPMI 1640 media (Wisent) supplemented with 10% FBS (Wisent), 1% penicillin/streptomycin (Wisent), 0.1 mM 2-mercaptoethanol (Bioshop), 10 mM HEPES, and 1 mM sodium pyruvate. Resting B cells were stimulated either with lipopolysaccharide (LPS; 5 μg/ml; Sigma-Aldrich) + IL-4 (5 ng/ml; PeproTech), LPS (25 μg/ml), or anti-CD40 (10 μg/ml, clone 1C10; eBioscience) + IL-4 (10 ng/ml; R&D Systems) + IL-5 (5 ng/ml; R&D Systems). Induced GCBCs (iGBs) were generated using 40LB feeder cells (a kind gift from Dr. Daisuke Kitamura, Tokyo University of Science, Tokyo, Japan; Nojima et al., 2011). 1 d before B cell plating, 40LB cells were irradiated (120 Gy) and plated at 0.3 × 10^6^ cells per well in 2 ml (6-well plate) or 0.13 × 10^6^ cells per well in 0.5 ml (24-well plate) DMEM media supplemented with 10% FBS (Wisent) and 1% penicillin/streptomycin (Wisent). Purified naive B cells were plated on 40LB feeders at 10^5^ cells per well in 4 ml of iGB media (6-well plate) or 2 × 10^4^ cells per well in 1 ml (24-well plate), supplemented with 1 ng/ml IL-4. At day 3 after plating, the same volume of fresh media was added to the wells, supplemented with 1 ng/ml IL-4 (PeproTech). On subsequent days, half of the volume per well was removed and replaced with media as above. For downstream applications, iGBs were purified by depleting 40LB using LS columns (cat. #130-042-401; Miltenyi Biotec) after incubation with biotinylated mAb against H2K^d^ (cat. #116303; BioLegend) at room temperature in 0.5% BSA, 2 mM EDTA PBS, followed by incubation with anti-biotin microbeads (cat. #130-105-637; Miltenyi Biotec) and purification using an iMag system (Nojima et al., 2011).

### Cell lines culture and treatments

The CH12F3 mouse BCL cell line, a kind gift from Dr. T. Honjo (Center for Cancer Immunotherapy and Immunobiology, Kyoto University, Kyoto, Japan; Nakamura et al., 1996), was cultured at 37°C with 5% (vol vol^−1^) CO_2_ in RPMI 1640 media (Wisent), supplemented with 10% FBS (Wisent), 1% penicillin/streptomycin (Wisent), and 0.1 mM 2-mercaptoethanol (Bioshop). Prmt1 was depleted by retroviral transduction of two independent shRNAs cloned in pLKO.1 (Sigma-Aldrich), #1 (TRCN000018491) and #2 (TRCN000018493; see Table S3). VSV-G, PAX2, and pLKO vectors (at 1:2.5:3.25 ratio, 1.35 μg DNA total) were transfected into HEK293 cells using Trans-IT LT-1 (MIR 2305; Mirus Bio). 2 d after transfection, 1 ml of HEK293 supernatant was added to 24-well plates coated with Retronectin (Takara) according to the manufacturer’s protocol and spun at 2,000 ×*g* for 90 min at 32°C. After removing the virus, 10^5^ CH12F3 cells were added per well in 1 ml and spun at 600 ×*g* for 30 min at 32°C. The next day, 1 ml of fresh media was added and 1 d later 1 μg/ml puromycin was added for the selection. RAJI and SU-DHL-5 cell lines, obtained from the American Type Culture Collection, were cultured in RPMI 1640 (Wisent), 10% FBS (Wisent), and 1% Pen/Strep (Wisent). RAJI were seeded at 0.5 × 10^6^ cells/ml and then kept between 0.2 × 10^6^ and 2 × 10^6^ cells/ml; SU-DHL-5 seeded at 0.1 × 10^6^ cells/ml and then kept between 0.1 × 10^6^ and 2 × 10^6^ cells/ml. The type I PRMT inhibitor, MS023 (cat#18361; Cayman Chemical), was resuspended at 50 mM in DMSO, aliquoted, and kept at −80 °C. Working dilutions in DMSO were kept at −20 °C and frozen/thawed up to three times for individual experiments. Cells were cultured with DMSO control or different doses of MS023 for 7 d and fed or split every 2–3 d depending on cell concentration. DMSO was 1/1,000 of the final culture volume. Rapamycin (cat. #R8781; Sigma-Aldrich) was prepared in DMSO and stored at −20 °C. Aliquots were thawed up to three times for experiments. P493-6 cells (a gift from Dr. T. Möröy, IRCM, Montreal, Canada) were cultured in RPMI 1640 (Wisent), 10% FBS tetracycline free (Thermo Fisher Scientific), 1% Pen/Strep (Wisent). Cells were seeded at 0.2 × 10^6^ cells/ml and kept at <2 × 10^6^ cells/ml. Doxycycline (cat. #DOX444; Bioshop) was resuspended at 1 mg/ml in distilled water and kept at −20°C. Cells were regularly checked for mycoplasma and validated by functional assays.

### Cell proliferation

Cell densities for growth curves of primary B cell derived iGBs were calculated by hemocytometer or using 123count eBeads (Invitrogen). Briefly, 200 μl of cells were mixed with 20 μl of beads and 5 μl propidium iodide (20 μg/ml); 1,000 beads were acquired by flow cytometry. To assess proliferation in vivo, 3 × 10^6^ splenocytes were first surface-stained for GC markers and then treated with fixation/permeabilization solution (cat. #00-5523; eBioscience) for 1 h at 4°C in the dark, washed twice in Perm buffer (eBioscience), followed by 1 h incubation with anti-Ki67-PECY7 (eBioscience) at 4°C and resuspended in PBS + 1% BSA. When necessary, anti-biotin staining was performed following Ki67 staining. Primary B cells in culture were stained with 1 μM CFSE (Invitrogen) on the day of plating, as described in the manufacturer’s protocol, and stimulated with cytokines. CH12 cells were stained with 5 μM CFSE (Invitrogen) 4 d after infection (2 d after puro selection), as described in the manufacturer’s protocol and stimulated with (1 μg/ml rat-antiCD40 [clone 1C10; eBioscience], 10 ng/ml IL-4 and 1 ng/ml TGFβ-1 [R&D Systems]) in the presence of 1 μg/ml puromycin to select for the shRNA vector.

### Apoptosis and cell cycle

To evaluate apoptosis in vivo, cells were incubated with FITC-conjugated CaspGLOW reagent that binds to all activated caspases (BioVision, K180). Briefly, 10^6^ cells were treated with 2 μl of FITC-VAD-FMK antibody for 1 h at 37°C in 300 μl warm media, washed, then surface stained and assessed immediately via flow cytometry. To assess apoptosis ex vivo 3–5 × 10^5^ cells were stained with 3 μl Annexin V-APC (cat. #550474; BD Pharmigen) in 100 μl of the provided binding buffer (×1) for 15 min at room temperature. Then 400 μl of binding buffer (×1) and 5 μl of propidim iodide (20 μg/ml) were added prior to flow cytometry acquisition. For cell cycle analysis, B cells were incubated with 10 μM BrdU for 1 h at 37°C in complete RPMI medium, then washed and resuspended in 200 μl cold PBS before fixing by adding the cells to pre-chilled 70% ethanol drop-wise under constant agitation and incubated on ice for 30 min. Then, 2 N HCl 0.5% Triton X-100 was added to the loosen cell pellet to denature the DNA, washed, resuspended in 0.1 M Na_2_B4O_7_, washed again and resuspended in PBS 0.5% Tween-20 1% BSA. Cells (10^6^) were then stained with anti-BrdU-FITC (1/50) for 30 min at room temperature in the dark before resuspending in PBS containing 5 μg/ml propidium iodide (#PPI888; Bioshop) and analyzed by flow cytometry. RAJI and SU-DHL-5 were treated for 24 h with rapamycin (Sigma-Aldrich) or palbociclib (#PZ0383; Sigma-Aldrich); and for P493-6 cells with doxycycline (Bioshop) or palbociclib. Cells harvested 24 h after treatment were fixed with cold (−20°C) 70% ethanol overnight. After 2 × 5 min PBS washes, cells were stained in 60 µg/ml propidium iodide, 0.5% Triton X-100 and 100 µg/ml RNAse A, in PBS during 15 min at room temperature in the dark. Finally, samples were washed with PBS, and data was acquired using the SA3800 Spectral Analyzer (Sony Biotechnology) and analyzed with FlowJo. RNA was extracted as described below, except that 3.5 µg of RNA purified from S2 cells (*Drosophila melanogaster*) were spiked into each cell sample after PBS wash for downstream normalization of RT-qPCR data. Cells were counted to ensure equal cell number per sample after PBS wash.

### Immunohistochemistry

Sections of 5-μm of paraffin-embedded tissues were deparaffinized in two changes of xylene for 5 min each and then rehydrated in distilled water using graded alcohols. Antigen retrieval was done by steaming the slides for 20 min then cooling for 20 min in EDTA buffer (1 mM EDTA, 0.05% Tween 20, pH 8) for AID and PRMT1. Endogenous peroxidase was blocked with a 0.3% hydrogen peroxide solution for 10 min. Endogenous biotin was blocked for 15 min with the blocking buffer provided with the Avidin/Biotin System (#SP2001; Vector Laboratories). For protein block, we used 10% normal goat serum and 1% BSA for 60 min at room temperature. Sections were incubated with anti-AID (1:50, rat Mab mAID-2 eBioscience), anti-PRMT1 (1:100) overnight at 4°C. Biotin-conjugated secondary antibodies were mouse anti–rabbit IgG (1:200; Vector Laboratories) to detect anti-PRMT1; mouse anti–rat IgG (1:200; Vector Laboratories) to detect anti-AID. Biotinylated reagents were detected with Vectastain ABC kit (PK-6100; Vector Laboratories). Peroxidase activity was developed using ImmPACT NovaRED HRP substrate (Vector Laboratories). Sections were counterstained with hematoxylin (cat. #MHS32-1L; Sigma-Aldrich) for 1 min prior to dehydrating and mounting for imaging on a bright field microscope.

### IF

Tissues were frozen in optimal cutting temperature compound (VWR #95057-838). Sections of 10 μm were fixed in paraformaldehyde 4% for 10 min at room temperature, washed three times in PBS at room temperature, followed by an incubation in pre-chilled acetone for 10 min at −20°C. Sections were permeabilized in 0.5% Triton X-100 in PBS for 10 min at room temperature, blocked in PBS 5% goat serum 1% BSA 0.3% Triton X-100 for 1 h at room temperature. Incubations with primary antibodies were performed in blocking solution overnight at 4°C in a humid chamber. When needed, secondary antibody was added in blocking solution for 1 h at room temperature in a humid chamber in the dark. Purified B cells were washed with PBS and then plated on coverslips coated with 0.1 mg/ml poly-L-lysine (Sigma-Aldrich). For single cell IF, cells were centrifuged 5 min at 400 ×*g*, then allowed to adhere at 37°C for 20 min, before fixation with 3.7% formaldehyde (Sigma-Aldrich) for 10 min at room temperature. After three washes with PBS, coverslips were blocked for 1 h with blocking solution (5% goat serum, 1% BSA, 0.5% Triton X-100 in PBS). Cells were then incubated overnight at 4°C with anti-aDMA ASYM26 antibody (1:500), diluted in blocking solution. After 3 × 5 min washes, with PBS + 0.1% Triton X-100 (PBS-T), cells were incubated for 1 h at room temperature with anti-rabbit IgG Alexa-546 (1:500) diluted in blocking solution. After 3 × 5 min washes with PBS-T, cells were incubated with 300 nM DAPI (Thermo Fisher Scientific) in PBS for 5 min at room temperature. Finally, coverslips were washed with PBS followed by ddH_2_O before mounting onto slides using Lerner Aqua-Mount (Thermo Fisher Scientific) before imaging. Antibodies are listed in Table S2.

### Microscopy

Images were acquired at room temperature using a Leica DM6 upright microscope (tissues), or a Zeiss LSM700 confocal microscope (tissues and single cells). For the LSM700, excitation lasers were 405 nM (DAPI and BV-421), 488 nM (FITC and Alexa488), 543 nM (R-PE and Alexa546), and 633 nM (Alexa680), with either 40×/1.3 or 63×/1.4 oil immersion objectives and collected using a Hamamatsu photomultiplier. Signal quantifications were done using Volocity (Perkin Elmer). For the DM6 microscope, the filter cubes used were DAPI, YFP, Cy3 and Cy5, with 20× objectives and collected using ORCAflash 4.0 V.2 from Hamamatsu, a high-resolution monochromatic camera. For each experiment, multiple fields were analyzed, excluding cells with saturated signal, abnormal DNA structure or mitotic figures. For figures, images were transferred to Photoshop for cropping, adjusting brightness and contrast for the whole image when necessary to enhance visibility.

### ELISPOT

Purified splenocytes or bone marrow (BM) cells were added at different dilutions to a 96-well 0.45 μm polyvinylidene difluoride membrane (cat. #MSIPS4W10; Millipore) previously coated overnight at 4°C with 2 μg/ml NP_20_BSA and blocked with complete RPMI cell culture media for 2 h at 37°C. Plates with cells were incubated in a humid chamber 12 h at 37°C, 5% CO_2_, then washed six times with PBS 0.01% Tween-20, followed by incubation with goat anti-mouse IgG1-HRP (A10551, 1/2,000; Life Technologies) diluted in culture media for 2 h at room temperature. Plates were washed and AEC substrate (3′ amino-9-ethylcarbazole; BD Bioscience) was added to reveal the spots. Images were acquired in an Axiophot MZ12 microscope and scored spots were counted from appropriate cell dilutions (2 × 10^6^ cells after primary immunization and 0.5 × 10^6^ cells for recall).

### ELISAs

Sandwich ELISA for measuring preimmune sera antibodies using anti-isotype–specific antibodies (BD Pharmingen) to capture IgM, IgG1, IgG2b, or IgG3 were done as described (Zahn et al., 2013). Antigen-specific antibodies were captured from immunized mice sera by coating ELISA plates with NP_20_-BSA (Biosearch Technologies) or CGG (100 ng/well; Biosearch Technologies) followed by the detection of IgG1, as described (Zahn et al., 2013). Sodium thiocyanate NaSCN displacement ELISA to measure antibody affinity/avidity was performed as described (Zahn et al., 2013), on plates coated as above. Sera were previously titrated by antigen-specific ELISA to choose a working dilution that ensured similar levels of antigen-specific antibodies across samples. Relative affinity values were calculated as described (Zahn et al., 2013).

### Western blotting

Cells were extracted in NP-40 lysis buffer (1% NP-40, 20 mM Tris, pH 8, 137 mM NaCl, 10% glycerol, 2 mM EDTA), containing protease and phosphatase inhibitor (Thermo Fisher Scientific). Extracts separated by SDS-PAGE were transferred to nitrocellulose membranes (Bio-Rad). Membranes were blocked in TBS 5% milk and probed with primary antibodies (1 h to overnight), washed 4 × 5 min in TBS 0.1% Tween-20 before incubating with secondary antibodies conjugated to AlexaFluor680 or IRDye800 for 1 h, washed and read on Odyssey CLx imaging system (LI-COR). Proteins were quantified using ImageStudiolite software. In some experiments, equal protein loading was controlled for by staining the membrane after transfer using either Ponceau-S or Revert total protein stain solution (LI-COR). The quantified Revert signal from a whole lane was used for normalization. Antibodies used for Western blotting are listed in Table S2.

### RT-PCR

RNA was isolated using TRIzol (Life Technologies) or TRI-reagent (Molecular Research Center, Inc), following manufacturer’s instruction, and quantified by NanoDrop (Thermo Fisher Scientific). cDNA was synthesized from 1 μg of RNA using the ProtoScript M-MuLV Taq RT-PCR kit and random primers (New England Biolabs). Quantitative PCR using SYBR select master mix (Applied Biosystems) was performed and analyzed in a ViiATM 7 machine and software (Life Technologies). Primers for quantitative PCR were obtained from literature or designed with NCBI RNA blast and synthesized at Integrated DNA technologies. For amplifying IGVH1-72/DJ rearrangements, RNA was isolated from sorted GCs of pools of two to three mice per genotype using RNAeasy Micro Kit (Qiagen) followed by an RT using SuperScript-IV RT (Thermo Fisher Scientific) as per the manufacturer’s instructions, except that RT was performed for 50 min. Heminested PCRs were done using KOD polymerase to amplify IgM (OJ794 and OJ796 followed by OJ794 and OJ795) or IgG1 (OJ794 and OJ2505 followed by OJ794 and OJ2506). Cycling conditions (95°C 2 min, 95°C 20 s, 55°C 10 s, and 70°C 10 s) × 40 cycles in the first PCR and 35 cycles in the second PCR. PCR products were purified by QIAquick PCR purification kit (Qiagen) and cloned in pGEM T-Easy vector (Promega). Minipreps using EZ-10 Spin Column Plasmid DNA Miniprep Kit (Biobasic) were sent for sanger sequencing to Genome Quebec CES. All primer sequences are listed in Table S3.

### SHM analysis

Sequences were trimmed in SnapGene (v5.3.2) to eliminate the PCR primers and inspected manually to confirm mutations and ensure the integrity of the sequencing. Duplicated sequences were considered PCR duplications and eliminated. FASTA files were submitted to IMGT/V-QUEST (https://www.imgt.org) for analysis. SHM frequency was calculated from sequences with in-frame junctions that mapped to the IGHV1-72*01 gene, and the frequency of clones carrying W33L, Y99G, and/or K59R mutations, which confer high affinity to NP (Allen et al., 1988; Furukawa et al., 1999), was calculated. The frequency of amino acid substitutions over the VH was plotted using a script described in (Chen et al., 2021).

### Transcriptome analyses

Gene expression data for Fig. 1, A and B were obtained from previously available B cell stages samples sorted from WT mice. Sample processing, preparation, and paired-end sequencing have been described (Kuchen et al., 2010). Additional data for activated B cell and GCBC were sequenced as single end with 50-bp read length. Reads were aligned to the mouse genome (mm9) with gsnap without detecting splice junctions de novo (--novelsplicing=0). Existing splice junctions from RefSeq annotation were considered (--use-splicing=/path/to/mm9.splices.iit). Output files were filtered to remove unaligned reads and any alignments with a mapping quality <20. Reads were mapped to RefSeq genes with htseq-count -m intersection-nonempty, and RPKM values were calculated from the counts. Density bed files were generated by using the bedtools genomecov program with a normalizing scale factor to calculate rpm and converted into bigwig files by using the UCSC toolkit bedGraphToBigWig. Data are available under accession number GSE112420.

RNA-seq from iGBs was performed on day 3.5 after plating purified splenic B cells from three mice each *Prmt1*^*F/F*^ Cγ1-cre and Cγ1-cre (two females and one male each) at 0.4 × 10^6^ cells/well with 1 ng/ml IL-4, in 6-well plates. Chromosome Y–specific transcripts were excluded from the analysis. After purifying iGBs by depleting 40LB cells, as described (Nojima et al., 2011), RNA was purified with RNeasy Plus Mini kit (Qiagen). RNA libraries and sequencing was performed at the IRCM facility as described (Litzler et al., 2019). Raw reads were trimmed using Trimmomatic v0.32 (Bolger et al., 2014). First, adaptors and other Illumina-specific sequences from each read were removed using palindrome mode. Then, a four-nucleotide sliding window removes the bases once the average quality within the window falls below 30. Next, the first four bases at the start of each read were removed. Finally, reads shorter than 30 base pairs were dropped. Cleaned reads were aligned to the mouse reference genome build mm10 using STAR v2.3.0e (Dobin et al., 2013) with default settings. Reads mapping to more than 10 locations in the genome (MAPQ < 1) were discarded. Gene expression levels were estimated by quantifying primary alignments mapping to at most two locations (MAPQ ≥ 3) to exonic regions (the maximal genomic locus of each gene and its known isoforms) using featureCounts v1.4.4 (Liao et al., 2014) and the mm10 ensGene annotation set from Ensembl. Normalization (mean of ratios), a variance-stabilized transformation of the data, and differential gene expression analysis were performed using DESeq2 v1.14.1 (Love et al., 2014). Data are available under accession number GSE189276. Gene expression changes in bulk RNA-seq data were analyzed by gene set enrichment analysis (GSEA; Subramanian et al., 2005), interrogating the Hallmark signature collection (Liberzon et al., 2015), or an ad hoc collection of signatures relevant to GCBC, listed in Table S1. Additional analyses of Gene Ontology terms defining Biological processes were performed using g:Profiler (https://biit.cs.ut.ee/gprofiler/gost). Signatures with a false discovery rate (FDRq-value) < 0.05 were considered significant.

### Genomic datasets

The following datasets of gene expression were mined as provided: microarray data GSE38304 (Dominguez-Sola et al., 2012), GSE38696 (Victora et al., 2012), GSE23925 (Victora et al., 2010), and GSE2350 (Basso et al., 2005). RNA-seq data GSE109732 (Ise et al., 2018), GSE98778 (Ersching et al., 2017), and GSE118124 (Andreani et al., 2018). Microarray data with associated survival data from GSE10846 (Lenz et al., 2008) and GSE93291 (Scott et al., 2017) were analyzed by log rank test using R2 (http://r2.amc.nl). Myc ChiP-seq samples from GSE80669 (Chou et al., 2016) and GSE51011 (Sabò et al., 2014) were processed using the ChIP-seq module of GenPipes v3.4.0 (Bourgey et al., 2019). Briefly, raw reads were trimmed using Trimmomatic v0.39 (Bolger et al., 2014), discarding reads shorter than 15 bp. Then, the resulting reads were aligned to the mm10 genome using bwa-mem v0.7.17 (Li and Durbin, 2010) with default parameters. Wiggle tracks are generated from aligned reads using Homer (Heinz et al., 2010) v4.11. Wiggle files from the same conditions have been merged by averaging the values using the “mean” parameter of wiggletools v1.2.5 and then converted to bigwig using wigToBigWig v4.

### scRNA-seq analysis

Raw gene expression counts for the following datasets were obtained, subjected to quality control, and processed using the Seurat package v4 (Hao et al., 2021): GSE148805 (Laidlaw et al., 2020), GSE162182 (Pae et al., 2021), GSE180920 (Duan et al., 2021), and VRJUNV (Roider et al., 2020). When possible, the processing steps followed the analysis workflow of the original reports

#### GSE148805 and GSE180920 datasets

Cells were filtered based on the following quality control metrics: cells with <200 feature counts or >4,000 feature counts, cells with >15,000 counts, and cells with >25% mitochondrial content. After filtering, 11,274 cells were left for analysis. Counts were scaled to 10,000 UMIs per cell and log-transformed. Dimensionality reduction was performed using principal component analysis (PCA) applied to the top 2,000 variable genes. The top 20 principal components were retained for graph-based clustering as implemented in Seurat (resolution 0.7) and for visualization using Uniform Manifold Approximation and Projection (UMAP). Gene markers for each cluster were inferred by differential gene expression analysis comparing cells from each cluster to all other cells in the sample, based on the Wilcoxon Rank-Sum test. Differentially expressed genes with a Bonferroni-adjusted P value < 0.05 were considered. Clusters were annotated based on the differential expression of canonical GCBC (*Bcl6*, *Cxcr4*, *Aicda*, *Cd83*, *Cd86*, and *Myc*), MBC (*Crr6*, *Cd38*, and *Hhex*), and naive B cell (*Ly6d*) markers. Naive follicular B cell clusters were filtered out, and dimension reduction, clustering (resolution = 0.35), and cluster labeling were performed again on the 7,691 remaining as described above. Finally, Myc^+^ B cells (defined as B cells with normalized *Myc* expression >1) were extracted and reclustered. Enrichment of gene signatures from the molecular signature database (MSigDB, Hallmark gene sets) and for post-GCBC (Duan et al., 2021) were computed based on single-sample GSEA (ssGSEA) as implemented in the escape package (Borcherding and Andrews, 2021) version 1.4.0.

#### GSE162182 dataset

Gene expression counts were normalized using the SCTransform function. Dimension reduction, clustering, and UMAP plots were performed as before using the top 30 principal components and a clustering resolution of 0.7. As in the original report, gene signature scores were calculated with the AddModuleScore function in Seurat for Myc targets, E2F targets, and mTORC targets. The same MSigDB gene sets employed by Pae et al. (2021) were used, namely SCHUHMACHER_MYC_TARGETS_UP, REN_BOUND_BY_E2F, and PENG_RAPAMYCIN_RESPONSE_DN. Signature scores were calculated for each time point at which GCBC were sorted. To analyze the kinetics of the aforementioned gene signatures, we selected the cells with a signature score >0.5 at each time point. In parallel, we analyzed the kinetics of *Prmt1* and *Myc* expression, by selecting the cells with normalized *Prmt1* or *Myc* expression >2 at each time point. For each gene or gene signature, the number of cells above the threshold at each time point was determined. The percentage of cells above the threshold was calculated as a fraction of the total number of cells above the threshold across all time points for each gene or gene signature.

#### VRJUNV dataset (Roider et al., 2020)

Four BCL samples from the dataset were analyzed: DLBCL1, DLBCL2, DLBCL3, and tFL1, filtering cells according to the characteristics of each sample based on the following quality control metrics. For DLBCL1, cells with >5,000 feature counts, cells with >40,000 counts, and cells with >10% mitochondrial content. For DLBCL2, cells were filtered based on the following quality control metrics: cells with >3,000 feature counts, cells with >15,000 counts, and cells with >10% mitochondrial content. For DLBCL3, cells were filtered based on the following quality control metrics: cells with >5,000 feature counts, cells with >30,000 counts, and cells with >25% mitochondrial content. For tFL1, cells were filtered based on the following quality control metrics: cells with >4,000 feature counts, cells with >20,000 counts, and cells with >10% mitochondrial content. After filtering, all four samples were integrated via Reciprocal PCA to generate a single heatmap with all cells from the samples. Using ssGSEA as implemented in the escape package, enrichment of gene signatures was computed as before with the following gene sets: HALLMARK_MYC_TARGETS_V1, HALLMARK_MYC_TARGETS_V2, HALLMARK_MTORC1_SIGNALING, GSE12366_GC_BCELL_VS_PLASMA_CELL_UP, GSE12366_GC_BCELL_VS_PLASMA_CELL_DN, and GSE12366_GC_VS_MEMORY_BCELL_DN. The ssGSEA signature scores were visualized with a heatmap, together with *MYC* and *PRMT1* expression levels across cells in all four samples. Cells were ordered according to *PRMT1* expression. The ssGSEA signature scores and gene expression values were also z-scaled normalized, and hierarchical clustering was performed on the rows using Euclidean distance as the distance measure. Next, the BCL samples were analyzed individually. Enrichment of gene signatures was again computed using ssGSEA. Cells in each sample were ordered by the normalized mean signature calculated using the following gene sets: HALLMARK_MYC_TARGETS_V1, HALLMARK_MTORC1_SIGNALING, as well as a GC signature gene set from GSE12366_GC_BCELL_VS_PLASMA_CELL_UP. Then, the levels of *PRMT1* expression were compared between the top and bottom 25% of cells ordered by the combined MYC/mTORC/GC signature.

### Statistics

Statistical analyses were performed using Prism 9.0 (GraphPad). Parametric methods were used for datasets in which all groups passed the Shapiro–Wilk normality test (α = 0.05). Otherwise, non-parametric tests were used. The specific statistical test used in each case is indicated in corresponding figure legends. P values were indicated in the figures with significance defined by α ≤ 0.05. Where no P value is indicated, it means that P > 0.05.

### Online supplemental material

Fig. S1 shows additional data for Prmt1 expression and analyses of *Prmt1*^*F/F*^ Cγ1-cre mice B cells. Fig. S2 shows additional data for the single-cell analysis of *Prmt1* expression in GCBC presented in Fig. 4 and the same analysis on a different data set, as well as additional information about SHM in Prmt1-null GCBC. Fig. S3 shows additional data on Prmt1 depletion in iGBs and CH12F3 B cells. Fig. S4 shows comparative data between Prmt1- and Prmt5-deficient B cells. Fig. S5 shows additional data about PRMT1 expression and the consequences of its inhibition in human BCL cells. Table S1 shows the MySigDB Hallmark signature collection. Table S2 lists antibodies for flow cytometry. Table S3 lists oligonucleotides primers for RT-qPCR.

## Supplementary Material

Table S1 shows the MySigDB Hallmark signature collection.

Table S2 lists antibodies for flow cytometry.

Table S3 lists oligonucleotides primers for RT-qPCR.

SourceData F1 contains original blots for Fig. 1.

SourceData F4 contains original blots for Fig. 4.

SourceData F8 contains original blots for Fig. 8.

SourceData FS3 contains original blots for Fig. S3.

SourceData FS5 contains original blots for Fig. S5.

## Data Availability

All flow cytometry analysis underlying any figure is available upon request. RNA-seq data underlying Fig. 1, A and B are openly available in National Center for Biotechnology Information at accession number GSE112420, and for Fig. 5, G and H and Fig. S3, I and J at accession number GSE189276. Accession numbers and references are provided in the article for all publicly available datasets analyzed. The IgV sequences used for SHM analyses were submitted to GenBank (accession numbers OQ915235 to OQ915340)
