# Addendum: Protein arginine methyltransferase 1 regulates B cell fate after positive selection in the germinal center in mice

**DOI:** 10.1084/jem.2022038111032025a

**Published:** 2025-11-10

**Authors:** Ludivine C. Litzler, Astrid Zahn, Kiersten L. Dionne, Adrien Sprumont, Silvana R. Ferreira, Michael R.F. Slattery, Stephen P. Methot, Anne-Marie Patenaude, Steven Hébert, Nisha Kabir, Poorani Ganesh Subramani, Seolkyoung Jung, Stéphane Richard, Claudia L. Kleinman, Javier M. Di Noia

Vol. 220, No. 9 | https://doi.org/10.1084/jem.20220381 | June 13, 2023

The authors wish to clarify an oversight regarding the reuse of a loading control blot in this publication. The article did not disclose that the actin blot presented in Fig. 1 D had been shown in a prior publication (Litzler et al., 2019, Fig. 1 b) because the blots in each publication are technical replicates from the same experiment involving B cell activation with LPS and IL-4. Fig. 1 from the *JEM* article is shown here for reference. This oversight does not affect the integrity of the data and the conclusions drawn from these experiments. The authors regret the error.

**Figure 1.**
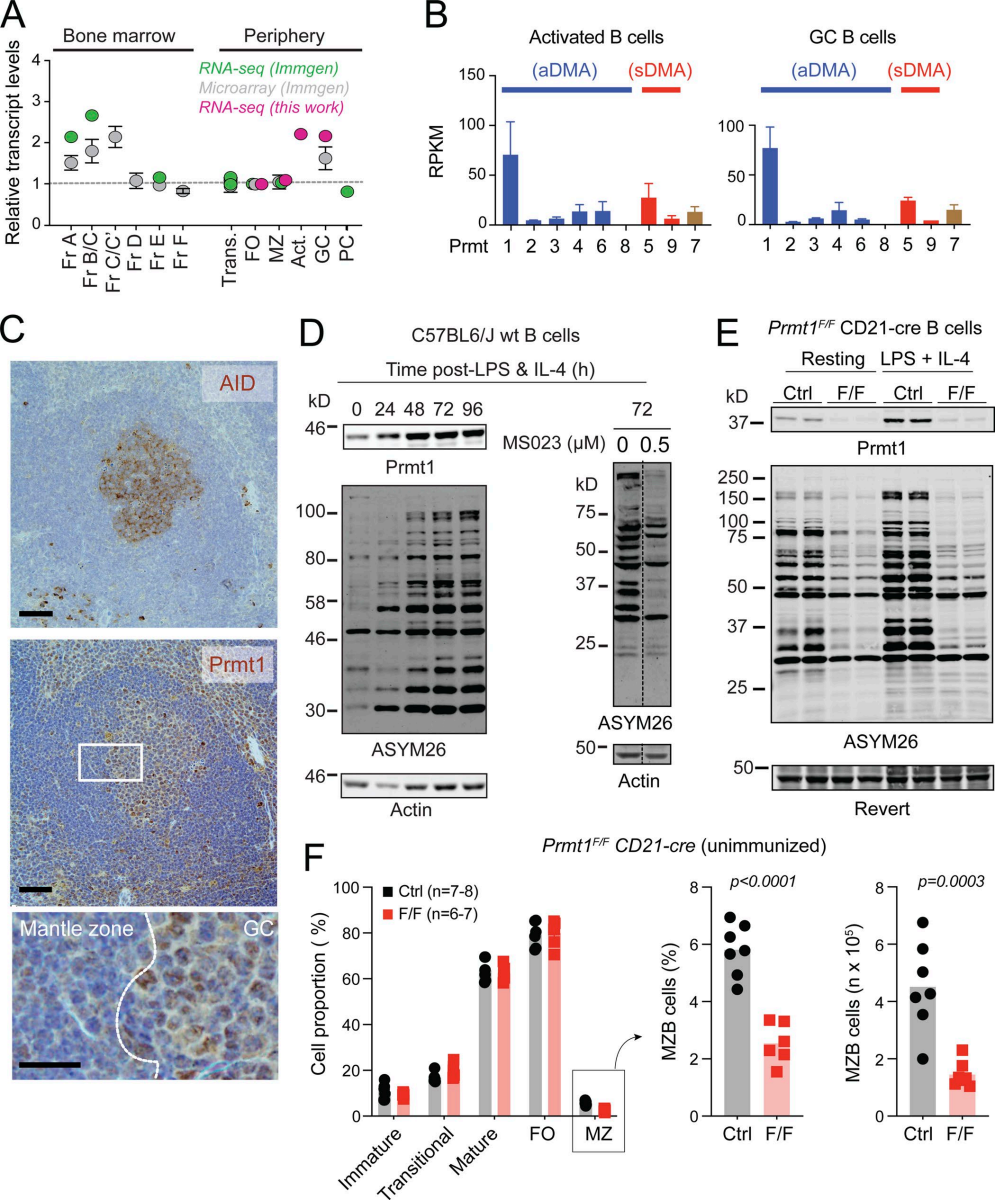
**Prmt1 expression in activated and GCBC. (A)** Prmt1 transcript levels in three mouse B cell datasets, each normalized to follicular (FO) B cells. Fr, Hardy’s fractions of B cell development; Trans, transitional; MZ, marginal zone; Act., ex vivo-activated mouse splenic B cells (50 µg/ml LPS + 2.5 ng/ml IL-4, 72 h). **(B)** PRMT transcript levels in activated (as in A) and in GCBC sorted from lymph nodes of immunized mice. Average + SEM RPKM from two biological replicates. **(C)** Immunohistochemistry for Prmt1, and AID as GC marker, on consecutive spleen sections from immunized mice. Representative of two mice/genotype independently analyzed. Scale bars, 100 µm (top, middle) and 20 µm (bottom). **(D)** Western blot of PRMT1, aDMA-modified proteins (ASYM26), and actin in extracts of resting and stimulated splenic B cells. MS023 = inhibitor of type I PRMTs. **(E)** Prmt1 and aDMA-proteins in extracts of resting or activated splenic B cells from two CD21-cre (Ctrl) and two *Prmt1*^*F/F*^ CD21-cre (F/F) mice. Revert protein stain as the loading control. **(F)** Proportion of splenic B cell subpopulations in individual *Prmt1*^*F/F*^*CD21-cre* (F/F) and *CD21-cre* (Ctrl) mice (symbols) from three independent experiments, with bars indicating means. MZB cell numbers are presented. P values by unpaired, two-tailed Student’s t test are indicated in the figure. Source data are available for this figure: SourceData F1.
