# Supplementary material for: Protein arginine methyltransferase 1 regulates B cell fate after positive selection in the germinal center in mice
Source: J Exp Med. 2023 Jun 13;220(9):e20220381. doi: 10.1084/jem.20220381 (PMC10266067; doi:10.1084/jem.20220381)
Supplement: Table S2 — lists antibodies for flow cytometry. [file JEM_20220381_TableS2.docx]

**Table S2. Antibodies**

**Antibodies for flow cytometry**

| Antigen | TAG | CATALOG | CLONE | VENDOR | DILUTION |
| --- | --- | --- | --- | --- | --- |
| **CD45R (B220)** | APC | 553092 | RA3-6B2 | BD Pharmingen | 1/200 |
| **CD45R (B220)** | PercP-Cy5.5 | 552771 | RA3-6B2 | BD Pharmingen | 1/200 |
| **CD45R (B220)** | Alexa Fluor 700 | 103232 | RA3-6B2 | BioLegend | 1/200 |
| **CD3e** | PE | 553063 | 145-2C11 | BD Pharmingen | 1/200 |
| **CD3e** | biotin | 553239 | 500-A2 | BD Pharmingen | 18 µl |
| **CD4** | biotin | 553045 | RM4-5 | BD Pharmingen | 18 µl |
| **IgD** | biotin | 13-5993-81 | 11-26c | eBioscience | 18 µl |
| **GL7** | BV421 | 562967 | GL7 | BD Pharmingen | 1/200 |
| **Fas (CD95)** | FITC | 554257 | Jo2 | BD Pharmingen | 1/200 |
| **Fas (CD95)** | BV421 | 562633 | Jo2 | BD Pharmingen | 1/200 |
| **Fas (CD95)** | biotin | 13-0951-85 | 15A7 | eBioscience | 1/50 |
| **CXCR4 (CD184)** | PE | 12-9991 | 2B11 | eBioscience | 1/100 |
| **CD86** | biotin | 13-0862 | GL1 | eBioscience | 1/100 |
| **Streptavidin** | BV605 | 405229 | - | BioLegend | 1/100 |
| **IgD** | FITC | 553439 | 11-26c.2a | BD Pharmingen | 1/200 |
| **IgD** | APC | 405713 | *11-26c.2a* | BioLegend | 1/200 |
| **IgM** | BV421 | 562595 | R6-60.2 | BD Pharmingen | 1/200 |
| **CD21/CD35** | APC | 558658 | 7G6 | BD Pharmingen | 1/200 |
| **CD23** | PE-Cy7 | 562825 | B3B4 | BD Pharmingen | 1/200 |
| **CD138** | APC | 561705 | 281-2 | BD Pharmingen |  |
| **CD138** | BV605 | 563147 | 281-2 | BD Pharmingen |  |
| **Ki67** | PE-Cy7 | 25-5698-82 | SolA15 | eBioscience | 1/1,000 |
| **IgG1** | PE | 550083 | A85-1 | BD Pharmingen |  |
| **IgG1** | biotin | 553441 | A85-1 | BD Pharmingen | 1/200 |
| **BrdU** | FITC | 347583 | B44 | BD Pharmingen | 1/50 |
| **BrdU** | FITC | MA1-12686 | *MoBU-1* | Invitrogen | 1/100 |
| **Annexin-V** | APC | 550474 | - | BD Pharmingen | 1/33 |
| **Pan activated Caspase (VAD-FMK inhibitor)** | FITC | K180 | - | BioVision | 1/50 |
| **IgA** | PE | 12-5994-81 | 11-44-2 | eBioscience |  |
| **CCR6** | PE-Cy7 | 129816 | 29-2L17 | Biolegend |  |
| **CD38** | FITC | 11-0381-82 | 90 | eBioscience | 1/100 |
| **CD38** | PE | 102708 | 90 | Biolegend | 1/300 |
| **CD80** | APC | 104713 | 16-10A1 | Biolegend | 1/40 |
|  |  |  |  |  |  |

**Antibodies for immunohistochemistry**

| AnTIGEN | TAG | CATALOG | CLONE | VENDOR | DILUTION | Raised in |
| --- | --- | --- | --- | --- | --- | --- |
| **PRMT1** | - | Ab3768 | - | Abcam | 1/100 | Rabbit |
| **AID** | - | 14-5959-82 | mAID-2 | eBioscience | 1/50 | Rat |
| **Rabbit-IgG** | biotin |  |  | Vector | 1/200 |  |
| **Rat-IgG** | biotin |  |  | Vector | 1/200 |  |

**Antibodies for immunofluorescence**

| AntiGEN | TAG | CATALOG | CLONE | VENDOR | DILUTION | Raised in |
| --- | --- | --- | --- | --- | --- | --- |
| **GL7** | BV421 | 562967 | GL7 | BD Pharmingen | 1/100 | Mouse |
| **IgD** | FITC | 553439 | 11-26c.2a | BD Pharmingen | 1/100 | Mouse |
| **ASYM26** | - | 13-0011 |  | EpiCypher | 1/500 | Rabbit |
| **Rabbit IgG** | Alexa 546 | A11010 |  | Invitrogen | 1/500 | Goat |
| **Rabbit IgG** | AlexaFluor 680 | A10043 |  | Invitrogen | 1/500 | Donkey |

**Antibodies for Western blots**

| AntiGEN | TAG | CATALOG | CLONE | VENDOR | DILUTION | Raised in |
| --- | --- | --- | --- | --- | --- | --- |
| **PRMT1** | - | MABE431 |  | Millipore | 1/2,000 | Rat |
| **ASYM26** | - | 13-0011 |  | EpiCypher | 1/1,000 | Rabbit |
| **Phospho-P70S6 Kinase1 (Thr389)** | - | 9206 | 1A5 | Cell Signaling Technology | 1/1,000 | Mouse |
| **P70S6 Kinase1** | - | 2708 | 49D7 | Cell Signaling Technology | 1/1,000 | Rabbit |
| **c-Myc** | - | 9402 |  | Cell Signaling Technology | 1/1,000 | Rabbit |
| **Rabbit IgG** | AlexaFluor 680 | A10043 |  | Invitrogen | 1/20,000 | Donkey |
| **Rat IgG** | AlexaFluor 680 | A21096 |  | Invitrogen | 1/20,000 | Goat |
| **Rabbit IgG** | IRDye800 | 925-32211 |  | LI-COR | 1/20,000 | Goat |
| **Mouse IgG** | IRDye800 | 926-32210 |  | LI-COR | 1/20,000 | Goat |
| **Actin** | - | A2066 |  | Sigma | 1/3,000 | Rabbit |
