# Supplementary material for: Protein arginine methyltransferase 1 regulates B cell fate after positive selection in the germinal center in mice
Source: J Exp Med. 2023 Jun 13;220(9):e20220381. doi: 10.1084/jem.20220381 (PMC10266067; doi:10.1084/jem.20220381)
Supplement: Table S3 — lists oligonucleotides primers for RT-qPCR. [file JEM_20220381_TableS3.docx]

**Table S3. Oligonucleotides**

**Primers for RT-qPCR**

| Gene | Type | Sequence (5’-3’) | Lab ID | Reference |
| --- | --- | --- | --- | --- |
| ***Prmt1*** | shRNA | CCGGGCTGAGGACATGACATCCAAACTCGAGTTTGGATGTCATGTCCTCAGCTTTTT | TRCN000018491 | (Sigma) |
| ***Prmt1*** | shRNA | CCGGGCAAGTGAAGAGGAACGACTACTCGAGTAGTCGTTCCTCTTCACTTGCTTTTT | TRCN000018493 | (Sigma) |
|  |  |  |  |  |
|  |  | ***Mouse qPCR*** |  |  |
| ***Prmt1*** | Forward | TTGGGATTGAGTGTTCCAGT | OJ867 |  |
|  | Reverse | TGCCCTTGATGATGGTCACC | OJ868 |  |
| ***Actin*** | Forward | CTCTGGCTCCTAGCACCATGAAGA | OJ897 |  |
|  | Reverse | GTAAAACGCAGCTCAGTAACAGTCCG | OJ898 |  |
| ***Bcl6*** | Forward | CACACCCGTCCATCATTGAA |  | Nurieva et al., 2008 |
|  | Reverse | TGTCCTCACGGTGCCTTTTT |  |  |
| ***Aicda*** | Forward | GCCACCTTCGCAACAAGTCT | OJ844 |  |
|  | Reverse | CCGGGCACAGTCATAGCAC | OJ845 |  |
| ***Prdm1*** | Forward | GGAGGATCTGACCCGAAT |  | Todd et al., 2009 |
|  | Reverse | TCCTCAAGACGGTCTGCA |  |  |
| ***Irf4*** | Forward | CTCTTCAAGGCTTGGGCATT |  | Todd et al., 2009 |
|  | Reverse | TGCTCCTTTTTTGGCTCCCT |  |  |
| ***Rpl35a*** | Forward | CGTGCCAAATTCCGAAGCAA | OJ1074 |  |
|  | Reverse | ATGGGTACAGCATCACACGG | OJ1075 |  |
| ***Igh*** | Forward | AGCTCACACCTTGACCTTTCA | OJ1621 | Minnich et al., 2016 |
|  | Reverse | TGGTGGGACGAACACATTTA | OJ1622 |  |
| ***Salpha*** | Forward | GGCTAGAATGGGCTAGAGTGAGTTA | OJ817 |  |
|  | Reverse | GCCTATTTTGGCCAGTCTACTTAC | OJ818 |  |
| ***Smu*** | Forward | TAGTAAGCGAGGCTCTAAAAAGCAC | OJ783 |  |
|  | Reverse | ACTCAGAGAAGCCCACCCAT | OJ784 |  |
| ***Bcl-2*** | Forward | GAGCGTCAACAGGGAGATGT | OJ2644 |  |
|  | Reverse | CTGGGGCCATATAGTTCCACAA | OJ2645 |  |
| ***Mcl-1*** | Forward | CCCCTCCCCCATCCTAATCA | OJ2646 |  |
|  | Reverse | CAATCCCTGGTCACTGTCGG | OJ2647 |  |
| ***Bcl-XL*** | Forward | AGTAAACTGGGGTCGCATCG | OJ2648 |  |
|  | Reverse | GCCATCCAACTTGCAATCCG | OJ2649 |  |
| ***Bax*** | Forward | CAGGATGCGTCCACCAAGAA | OJ2650 |  |
|  | Reverse | AGTCCGTGTCCACGTCAGCA | OJ2651 |  |
| ***Bad*** | Forward | CAGCAGCCCAGAGTATGTTCC | OJ2652 |  |
|  | Reverse | CGTCCCTGCTGATGAATGTTG | OJ2653 |  |
| ***c-Myc*** | Forward | GCG​ACT​CTG​AAG​AAG​AGC​AAG | OJ2682 |  |
|  | Reverse | GCC​TCG​GGA​TGG​AGA​TGA​G | OJ2683 |  |
| ***VH-IgM*** | Forward | TCTTCTTGGCAGCAACAG | OJ794 | For SHM on IGHV1-72 IgM |
|  | Reverse | AGGTTCTGATACCCTGGATGAC | OJ796 |  |
|  |  | CCAGATTCTTATCAGACAGG | OJ795 |  |
| ***VH-IgG1*** | Forward | TCTTCTTGGCAGCAACAG | OJ794 | For SHM on IGHV1-72 IgG1 |
|  | Reverse | GGAAGGTGTGCACACCGCTGGAC | OJ2505 |  |
|  |  | GCTCAGGGAAATAGCCCTTGAC | OJ2506 |  |
|  |  |  |  |  |
|  |  | ***Human qPCR*** |  |  |
| ***ACTIN*** | Forward | TCCCTGGAGAAGAGCTACGA | OJ1012 |  |
|  | Reverse | AGCACTGTGTTGGCGTACAG | OJ1013 |  |
| ***PRMT1*** | Forward | TTGGGATTGAGTGTTCCAGT | OJ867 |  |
|  | Reverse | TGCCCTTGATGATGGTCACC | OJ868 |  |
| ***PRDM1*** | Forward | GACGAAGCGAGGAGGGAC | OJ1883 |  |
|  | Reverse | GGGGCAGCCAAGGTCGTA | OJ1884 |  |
| ***IRF4*** | Forward | TCCCAGCCCAGGTTCACAAC | OJ1885 |  |
|  | Reverse | CAGGTGGGGCACAAGCATA | OJ1886 |  |
| ***XBP1*** | Forward | CTGAGTCCGCAGCAGGTG | OJ1887 |  |
|  | Reverse | GTCCAGAATGCCCAACAGGA | OJ1888 |  |
| ***BCL6*** | Forward | CAGTCCCCACTCACTCACAT | OJ1889 |  |
|  | Reverse | AGGCCATTTTGTCTTCACCAAT | OJ1890 |  |
| ***PAX5*** | Forward | CACCATGTTTGCCTGGGAGA | OJ1891 |  |
|  | Reverse | CGGAGCCAGTGGACACTATG | OJ1892 |  |
| ***C-MYC*** | Forward | GCCACGTCTCCACACATCAG | OJ2678 |  |
|  | Reverse | TGGTGCATTTTCGGTTGTTG | OJ2679 |  |
|  |  |  |  |  |
|  |  | ***Drosophila qPCR*** |  |  |
| ***Act5c*** | Forward | AAGTTGCTGCTCTGGTTGTCG | OJ2281 | Fitz et al., 2020 |
| ***Act5c*** | Reverse | GCCACACGCAGCTCATTGTAG | OJ2282 |  |

**References**

Fitz, J., T. Neumann, M. Steininger, E.-M. Wiedemann, A.C. Garcia, A. Athanasiadis, U.E. Schoeberl, and R. Pavri. 2020. Spt5-mediated enhancer transcription directly couples enhancer activation with physical promoter interaction. *Nat. Genet.* 52:505–515. https://doi.org/10.1038/s41588-020-0605-6

Minnich, M., H. Tagoh, P. Bönelt, E. Axelsson, M. Fischer, B. Cebolla, A. Tarakhovsky, S.L. Nutt, M. Jaritz, and M. Busslinger. 2016. Multifunctional role of the transcription factor Blimp-1 in coordinating plasma cell differentiation. *Nat. Immunol.* 17:331–343. https://doi.org/10.1038/ni.3349

Nurieva, R.I., Y. Chung, D. Hwang, X.O. Yang, H.S. Kang, L. Ma, Y.-h. Wang, S.S. Watowich, A.M. Jetten, Q. Tian, and C. Dong. 2008. Generation of T follicular helper cells is mediated by interleukin-21 but independent of T helper 1, 2, or 17 cell lineages. *Immunity*. 29:138–149. https://doi.org/10.1016/j.immuni.2008.05.009

Todd, D.J., L.J. McHeyzer-Williams, C. Kowal, A.-H. Lee, B.T. Volpe, B. Diamond, M.G. McHeyzer-Williams, and L.H. Glimcher. 2009. XBP1 governs late events in plasma cell differentiation and is not required for antigen-specific memory B cell development. *J. Exp. Med.* 206:2151–2159. https://doi.org/10.1084/jem.20090738
