# Supplementary figures and images for: Protein arginine methyltransferase 1 regulates B cell fate after positive selection in the germinal center in mice
Source: J Exp Med. 2023 Jun 13;220(9):e20220381. doi: 10.1084/jem.20220381 (PMC10266067; doi:10.1084/jem.20220381)

Figure 1D

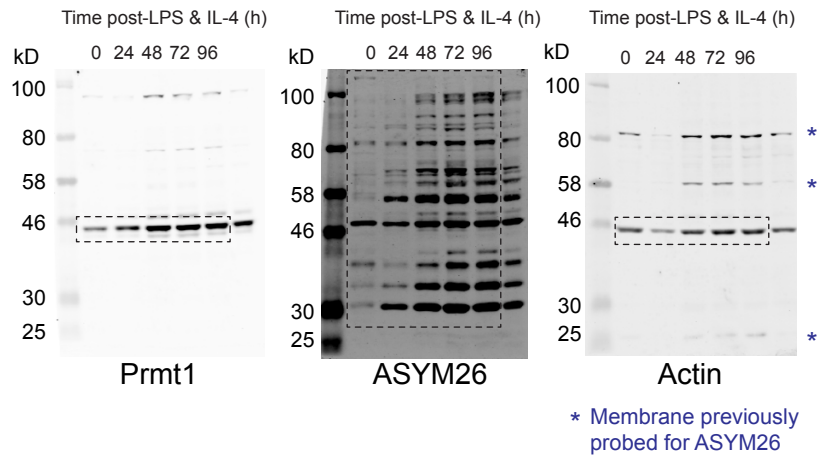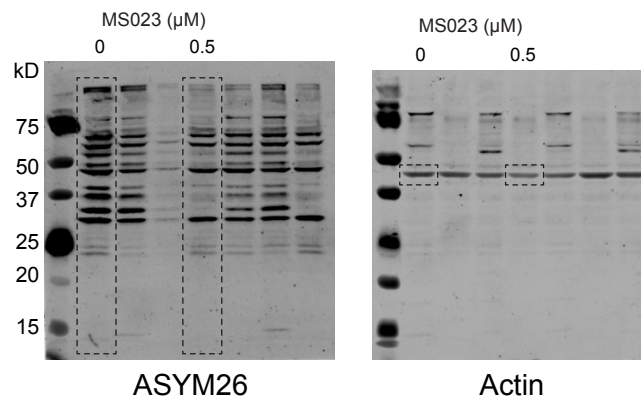

Figure 1E

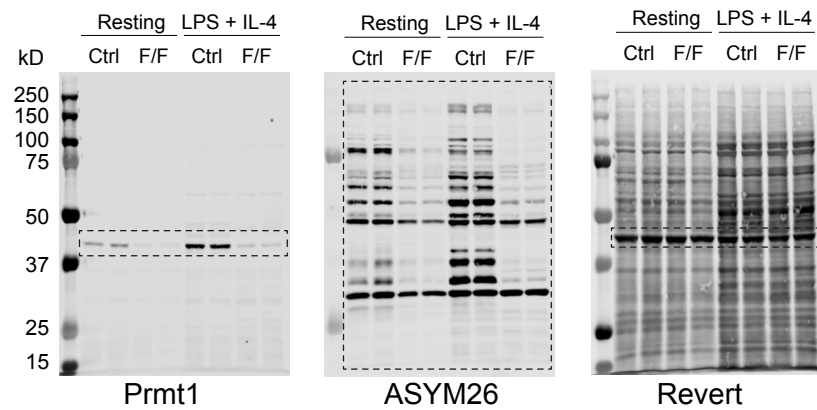

Supplement: SourceData F1 — contains original blots for Fig. 1. [file JEM_20220381_SourceDataF1.pdf]

Figure 4F

Gel1

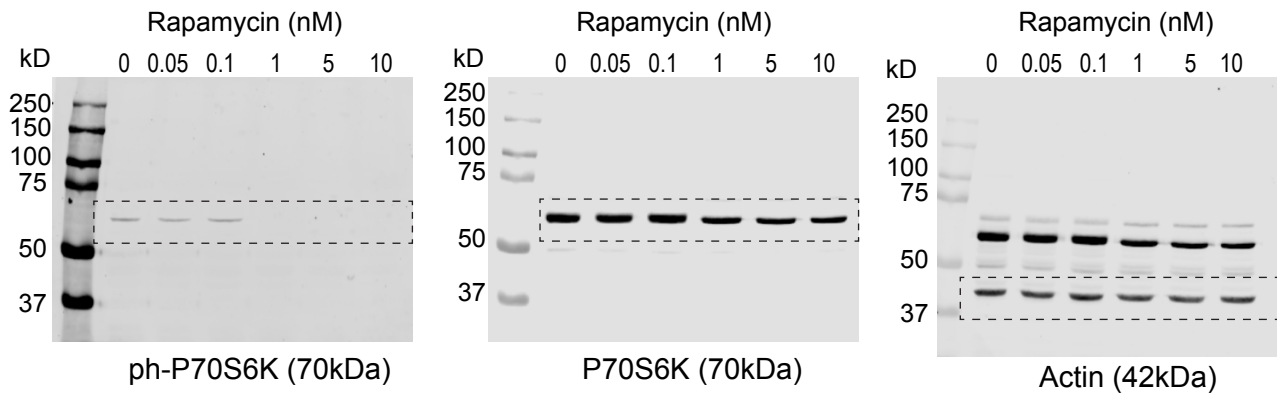

Gel2

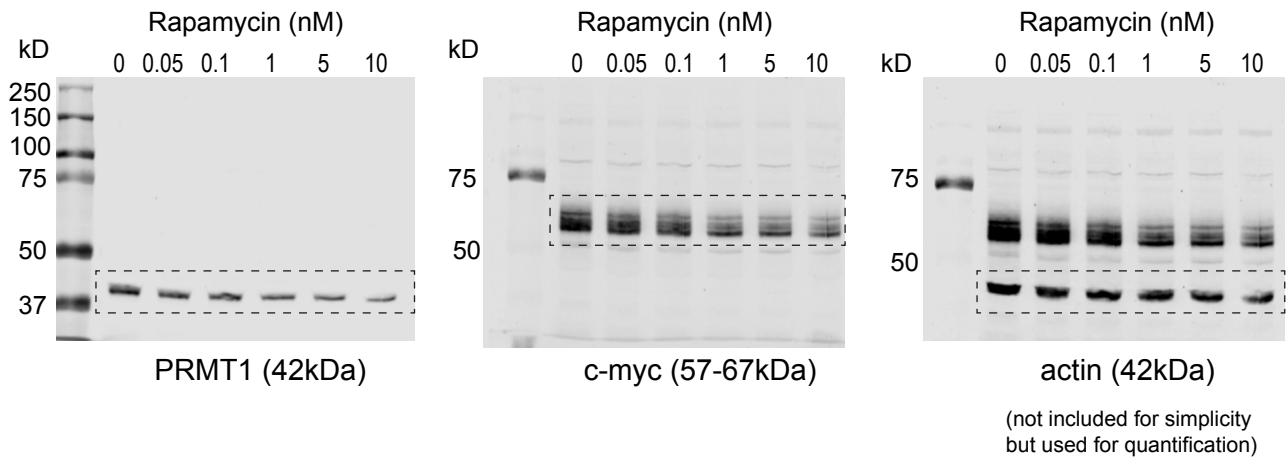

Supplement: SourceData F4 — contains original blots for Fig. 4. [file JEM_20220381_SourceDataF4.pdf]

Figure 8D

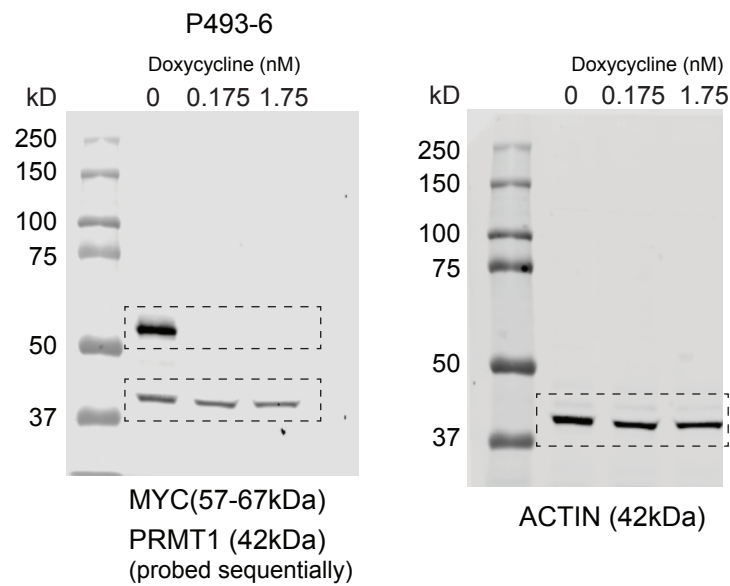

Figure 8E

Gel1

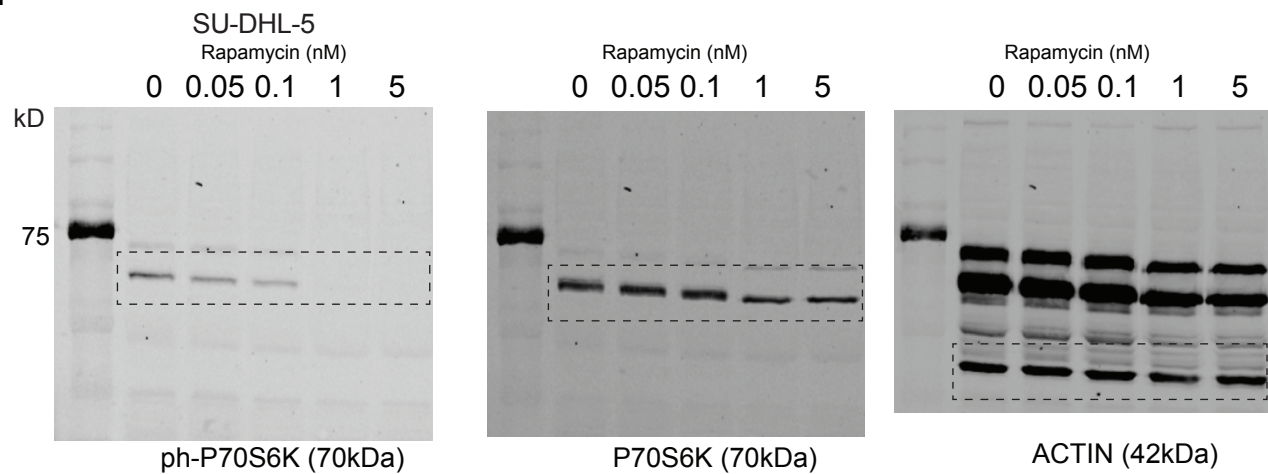

Gel2

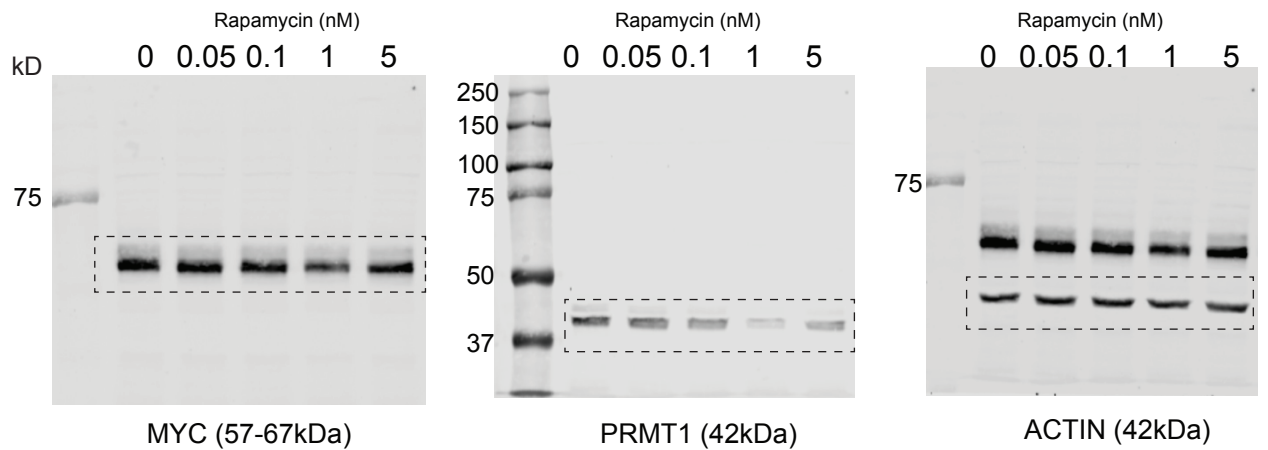

Supplement: SourceData F8 — contains original blots for Fig. 8. [file JEM_20220381_SourceDataF8.pdf]

Figure S3C

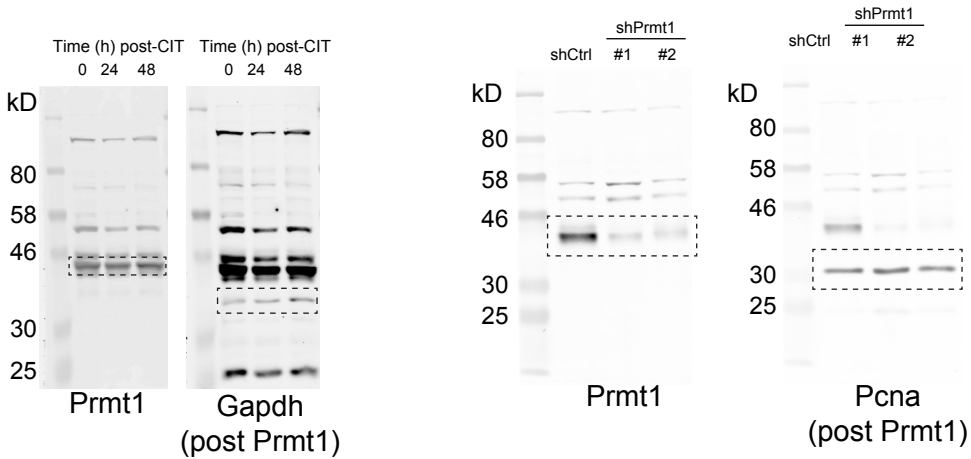

Figure S3D

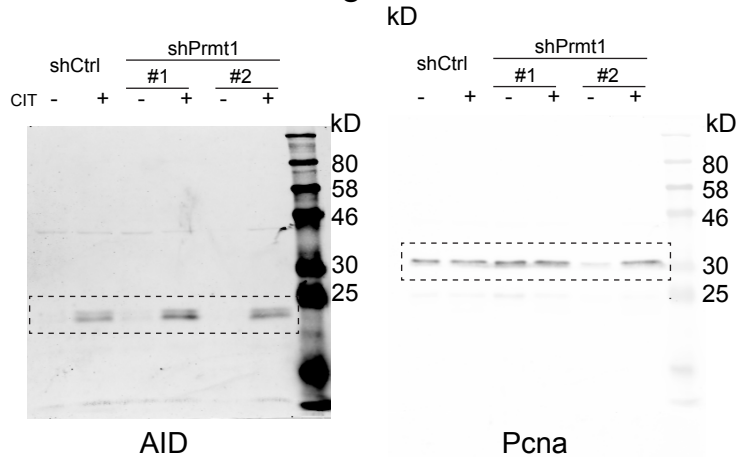

Supplement: SourceData FS3 — contains original blots for Fig. S3. [file JEM_20220381_SourceDataFS3.pdf]

Figure S5J

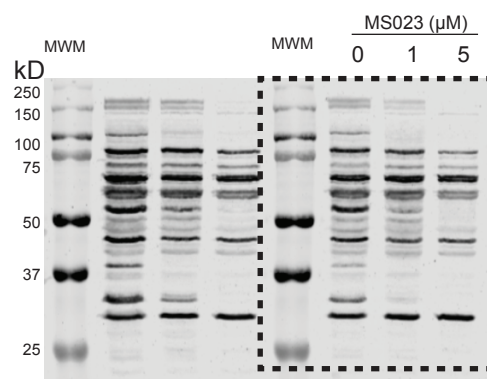

Supplement: SourceData FS5 — contains original blots for Fig. S5. [file JEM_20220381_SourceDataFS5.pdf]
